# Thyroid dysfunction after immune checkpoint inhibitors in a single-centre UK pan-cancer cohort: A retrospective study

**DOI:** 10.1016/j.ejca.2024.113949

**Published:** 2024-02-21

**Authors:** Oliver John Kennedy, Nadia Ali, Rebecca Lee, Phillip Monaghan, Safwaan Adam, Tim Cooksley, Paul Lorigan

**Affiliations:** a The Christie NHS Foundation Trust, Manchester, M20 4BX, UK; b Division of Cancer Sciences, The University of Manchester, Manchester, M13 9PL, UK; c The Christie Pathology Partnership, Manchester, M20 4BX, UK

**Keywords:** Immune checkpoint inhibitors, Immunotherapy, Thyroiditis, Hyperthyroidism, Hypothyroidism, Overall survival

## Abstract

**Purpose:**

This study investigated thyroid dysfunction with immune checkpoint inhibitors (ICIs) in terms of proportions affected, risk factors, thyroid sequelae, and overall survival (OS).

**Methods:**

Among patients with normal baseline free T4 (fT4) and thyroid stimulating hormone (TSH) receiving ICIs at a large cancer centre, proportions of hyperthyroidism/hypothyroidism were determined (any, subclinical [normal fT4, abnormal TSH], overt [abnormal fT4, abnormal TSH], isolated hyperthyroxinaemia/hypothyroxinaemia and secondary) with onset times and subsequent thyroid statuses. Associations of overt dysfunction with OS were estimated using Cox regression and methods robust to immortal time bias (time-dependent Cox regression and 3- and 6-month landmark analyses). Associations of baseline variables with overt hyperthyroidism and hypothyroidism were estimated using Fine and Gray regression.

**Results:**

Of 1349 patients, 34.2% developed hyperthyroidism (10.3% overt), including 54.9% receiving combination ICIs, while 28.2% developed hypothyroidism (overt 9.3%, secondary 0.5%). A third of overt hypothyroidism cases occurred without preceding hyperthyroidism. Subclinical thyroid dysfunction returned directly to normal in up to half. Overt hyperthyroidism progressed to overt hypothyroidism in 55.4% (median 1.6 months). Melanoma treatment in the adjuvant vs. advanced setting caused more overt hyperthyroidism (12.1% vs. 7.5%) and overt hypothyroidism (14.5% vs. 9.7%). Baseline eGFR < 60 mL/min/1.73 m^2^ (HR=1.68, 1.07–2.63) was associated with overt hyperthyroidism and sex (HR=0.60, 0.42–0.87) and TSH (4th vs. 1st quartile HR=1.87, 1.10–3.19) with overt hypothyroidism. Overt dysfunction was associated with OS in the Cox analysis (HR=0.65, 0.50–0.85, median follow-up 22.2 months) but not in the time-dependent Cox (HR=0.79, 0.60–1.03) or landmark analyses (3-month HR=0.74, 0.51–1.07; 6-month HR=0.91, 0.66–1.24).

**Conclusion:**

Thyroid dysfunction affects up to half of patients receiving ICIs. The association with OS is unclear after considering immortal time bias. The clinical courses include recovery, thyrotoxicosis and de novo overt hypothyroidism. Adjuvant treatment for melanoma, where longer-term harms are of concern, causes more frequent/aggressive dysfunction.

## 1 Introduction

Immune checkpoint inhibitors (ICIs) have markedly improved outcomes in multiple cancers [1]. While first approved in the advanced setting, ICIs are now used widely for adjuvant and neoadjuvant treatment of earlier stage cancers [2–4]. In earlier stage disease, however, the benefits are less clear, particularly regarding overall survival (OS), yet the potential for longer-term harms from immune-related adverse events (irAEs) may be greater. One of the most common irAEs is thyroid dysfunction [3,5–9]. Previous studies have linked ICI-induced thyroid dysfunction to progression-free survival (PFS) [10] and, in the advanced setting, overall survival (OS) [11]. However, previously reported associations have frequently not accounted for ‘immortal time bias’ between treatment initiation and thyroid dysfunction onset. Aside from its potential association with OS, thyroid dysfunction also has negative implications. In the general population, non-ICI induced thyroid disorders are associated with a 3–4 year reduction in life expectancy [12], reduced quality of life [13] and increased death rates from cardiovascular and cerebrovascular diseases [14,15].

Reports differ regarding the proportions affected by ICI thyroid dysfunction as well as the severity, duration (temporary vs. permanent) and patterns of dysfunction (e.g. initial hyperthyroidism followed by hypothyroidism) [2–4,7,16–20]. Previous analyses of risk factors have not always accounted for competing risks [10,19], and the effect of dysfunction on OS is unclear regarding timing of onset and severity. There may be variations in the characteristics of thyroid dysfunction for different ICIs, cancers and treatment intents (e.g. adjuvant vs. advanced). Given these uncertainties and the potential impact of thyroid disorders on patients, this study aimed to characterise the relationship between ICIs and thyroid dysfunction in a large real-world cohort.

## 2 Methods

Patients were treated between January 2018 and September 2022 at The Christie NHS Foundation Trust, Manchester, UK, a large centre serving an ethnically diverse population. Patients were included if they (i) received an ICI for any cancer; (ii) had normal baseline free T4 (fT4) and thyroid stimulating hormone (TSH) levels; and (iii) had ≥ 1 measurement of fT4 and TSH during follow-up. Patients treated previously with an ICI were excluded.

Thyroid function tests were, in general, performed before treatments, at clinic appointments (usually not less than 3 monthly), during acute admissions and whenever any irAE was suspected. After treatment discontinuation, patients usually remained under follow-up and around 60% had continuing thyroid assessments. Details of assays and reference ranges are provided as supplementary information.

Crude hyperthyroidism proportions were calculated according to: (i) high fT4 and low TSH (i.e. “overt primary hyperthyroidism”); (ii) high fT4 and normal TSH (i.e. isolated hyperthyroxinaemia); (iii) normal fT4 and low TSH (i.e. “subclinical hyperthyroidism”); and (iv) high fT4 and high TSH (i.e. “overt secondary hyperthyroidism”). Separately, crude hypothyroidism proportions were calculated according to: (i) low fT4 and high TSH (i.e. “overt primary hypothyroidism”); (ii) low fT4 and normal TSH (i.e. isolated hypothyroxinaemia); (iii) normal fT4 and high TSH (i.e. “subclinical hypothyroidism”); and (iv) low fT4 and low TSH (i.e. “overt secondary hypothyroidism”). Secondary dysfunction was confirmed by review of subsequent investigations. Proportions were estimated according to diagnosis, ICIs, adjuvant vs. advanced setting (melanoma only) and renal cancer vs. melanoma for combination ipilimumab/nivolumab. Median times to overt hyperthyroidism and hypothyroidism from ICI initiation were also estimated.

Proportions of patients with different types of thyroid dysfunction at onset who returned directly to normal vs. progressed to other thyroid dysfunction states were calculated. Among those with overt hypothyroidism, proportions with and without preceding hyperthyroidism were determined along with onset times relative to the preceding normal blood test. Trajectories of fT4 and TSH were examined in a subset of patients with comprehensive follow-up (i.e. minimum of 6-weekly blood tests) and who developed overt hyperthyroidism followed by overt hypothyroidism. This was to illustrate changes in thyroid hormones in aggressive ICI-induced dysfunction. In this group, daily fT4 and TSH values were estimated using linear interpolation, and the daily medians across all patients were plotted after smoothing with a Gaussian filter.

Hazard ratios (HRs) and 95% confidence intervals (CIs) for associations of baseline variables (sex, age, body mass index [BMI], estimated glomerular filtration rate [eGFR], aspartate aminotransferase [AST], neutrophil lymphocyte ratio [NLR], lactate dehydrogenase [LDH] and TSH) with cumulative incidences of overt hyperthyroidism and overt hypothyroidism (separately) were estimated in Fine and Gray analyses. Death was included as a competing risk since this precluded thyroid testing. Adjustments were included for age, sex, performance status (PS), ICI, setting (advanced vs. adjuvant) and cancer type. Non-cases were right-censored at data extraction, with the most recent thyroid function forward-extrapolated. Cumulative incidence curves were also estimated. Sensitivity analyses were performed to investigate the influence of treatment discontinuation. Specifically, associations were estimated within the first 6 months of follow-up (with right-censoring at the end of this period) among patients who did not discontinue treatment in that time. A period of 6 months was selected for balance between sufficient time for thyroid dysfunction to occur and sample size.

HRs for associations between the presence vs. absence of [i] overt thyroid dysfunction, [ii] overt hyperthyroidism, and [iii] overt hypothyroidism with OS were estimated using Cox regression, similarly to previous studies. Associations were also estimated using methods that account for immortal time bias: time-dependent Cox regression (i.e. with a time-varying covariate for thyroid dysfunction with a value of 0 and 1, respectively, before and after onset) and 3- and 6-month landmark analyses [21]. In the landmark analyses, patients were excluded if they were censored or died before the landmark, and exposure assignment was according to thyroid dysfunction occurring any time pre-landmark. Two landmarks of 3 and 6-months were selected based on previously reported median onset times [19]. Survival curves were generated using the Kaplan-Meier method. A time-dependent Cox model was also used to test for interactions of cancer type and ICI with the overt dysfunction-OS association. Non-cases were right-censored at data extraction.

In the Cox and landmark analyses, thyroid function was also considered as a variable with multiple categories: no thyroid dysfunction, subclinical hyperthyroidism, subclinical hypothyroidism, overt hyperthyroidism vs. de novo overt hypothyroidism. The same adjustments were made as above. Finally, associations of treatment indications (a combination of ICI, setting and cancer type) with OS were estimated using Cox regression, and associations with overt hyperthyroidism and overt hypothyroidism were estimated using Fine and Gray regression. Both analyses were adjusted for age, sex and PS.

Analyses were performed in Python 3.11.2 and R 4.3.0. The Reporting of studies Conducted using Observational Routinely-collected Data (RECORD) statement was followed [22]. This study was approved by the Quality Improvement and Clinical Audit Committee at the Christie NHS Foundation Trust (reference 3180).

## 3 Results

Table 1 and supplementary table 1 detail baseline characteristics of the 1349 included patients. The majority were male (63.5%), aged ≥ 60 years (65.2%), and had an Eastern Cooperative Oncology Group (ECOG) performance status of 0 (57.3%) or 1 (26.7%). More patients were treated in the advanced (N = 1151) than adjuvant setting (N = 198), where pembrolizumab or nivolumab for melanoma (N = 124) and durvalumab for lung cancer (N = 35) were commonest. The median number of thyroid function measurements was 8 (supplementary figure 1) and median time until the final measurement was 8.2 months (interquartile range=3.2–15.8 months, supplementary figure 2).

### 3.1 Proportions with thyroid dysfunction, time to onset and severity

In total, 462 patients (34.2%) developed hyperthyroidism (subclinical 18.8%; overt 10.3%; isolated hyperthyroxinaemia 5.0%) (Table 2), including two patients with overt secondary hyperthyroidism. Both those patients had concurrent irAEs (not directly affecting the thyroid or pituitary), and fT4 and TSH were only marginally above the reference range and normalised within two weeks. Combination ipilimumab/nivolumab had the highest proportions of hyperthyroidism (any 54.9%, overt 22.3%). Atezolizumab had the lowest (any 25.2%, overt 3.0%). Renal cancer was the most affected (any 50.8%, overt 22.0%), and lung cancer the least. The median time to overt hyperthyroidism was 1.4 months. The median peak fT4 in overt hyperthyroidism was 28.5 pmol/L, and the maximum was 144.8 pmol/L (combination ipilimumab/nivolumab).

Three hundred and eighty-one patients developed hypothyroidism (any 28.2%, subclinical 16.2%, overt 9.3%, isolated hypothyroxinaemia 2.1%), including seven (0.5%) with overt secondary hypothyroidism from hypophysitis or, potentially in one case, non-thyroidal illness (Table 3). Combination ipilimumab/nivolumab had the highest proportions (any 41.9%, overt 14.0%). Chemoimmunotherapy had the lowest proportion of any hypothyroidism (15.5%) and atezolizumab had the lowest rate of overt hypothyroidism (3.7%). The median time to overt hypothyroidism was 3.4 months (i.e. later than overt hyperthyroidism), ranging from 3.2 months for nivolumab to 4.1 months for atezolizumab.

### 3.2 Thyroid dysfunction in melanoma by treatment intent and in ipilimumab/nivolumab by diagnosis

Among patients with melanoma, 124 received adjuvant treatment (pembrolizumab n = 98; nivolumab n = 26) compared to 93 with advanced disease (pembrolizumab n = 71; nivolumab n = 22). Adjuvant treatment caused more hyperthyroidism (any 35.5% vs. 30.1%; overt 12.1% vs. 7.5%) with higher median peak fT4 (30.6 vs. 27.5 pmol/L) and maximum fT4 (68.8 vs. 40.3 pmol/L). It resulted in more hypothyroidism (any 25.8% vs. 20.4%, overt 14.5% vs. 9.7%) with faster onset (first low fT4 3.2 vs. 4.4 months). 103 melanoma patients received ipilimumab/nivolumab compared to 101 with renal cancer. Hyperthyroidism occurred more commonly in renal cancer (any 60.4% vs. 50.5%, overt 24.8% vs. 20.4%). In melanoma, however, the median peak fT4 was higher (38.4 vs. 28.5 pmol/L). Proportions of overt hypothyroidism were similar (14.6% vs 13.9%) with a shorter onset in melanoma (2.7 vs. 4.3 months).

### 3.3 Patterns of thyroid dysfunction

**Fig. 1:**
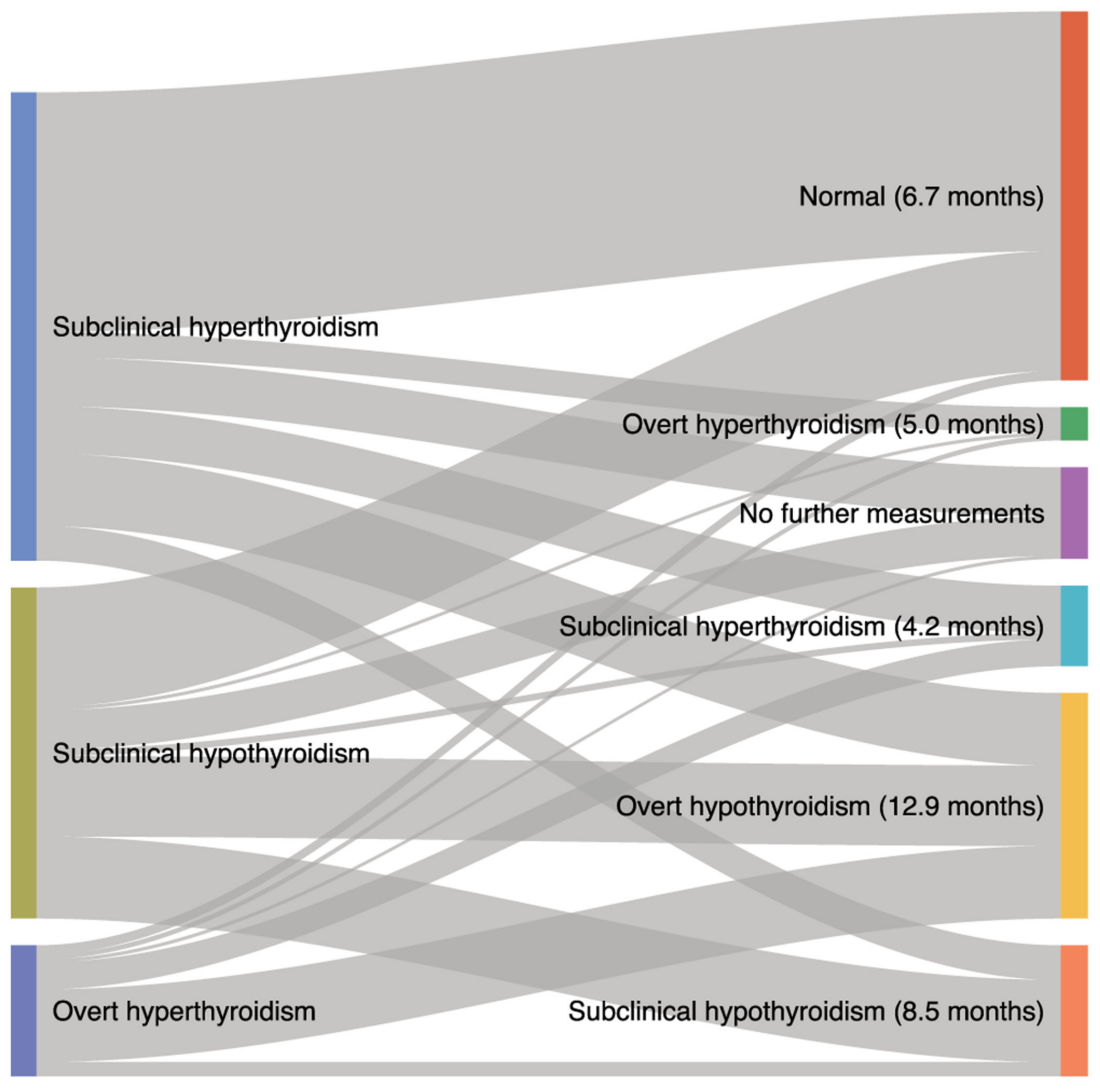
A Sankey plot showing patients who, after their first detected thyroid dysfunction, returned directly to normal thyroid function vs. progressed to other states of dysfunction (median times between first and last TFT).

Figure 1 illustrates the proportions of patients with different types of thyroid dysfunction at onset who returned directly to normal vs. progressed to other states of dysfunction. After subclinical hyperthyroidism, 51.2% returned directly to normal thyroid function, while 15.5% and 5.4%, respectively, developed overt hypothyroidism or overt hyperthyroidism. Following subclinical hypothyroidism, 36.2% returned directly to normal, while 24.3% developed overt hypothyroidism. After overt hyperthyroidism, 55.4% developed overt hypothyroidism, 7.2% returned directly to normal, while the remainder mostly developed subclinical dysfunction. Follow-up was longer in patients who developed overt hypothyroidism (supplementary table 2).

**Fig. 2:**
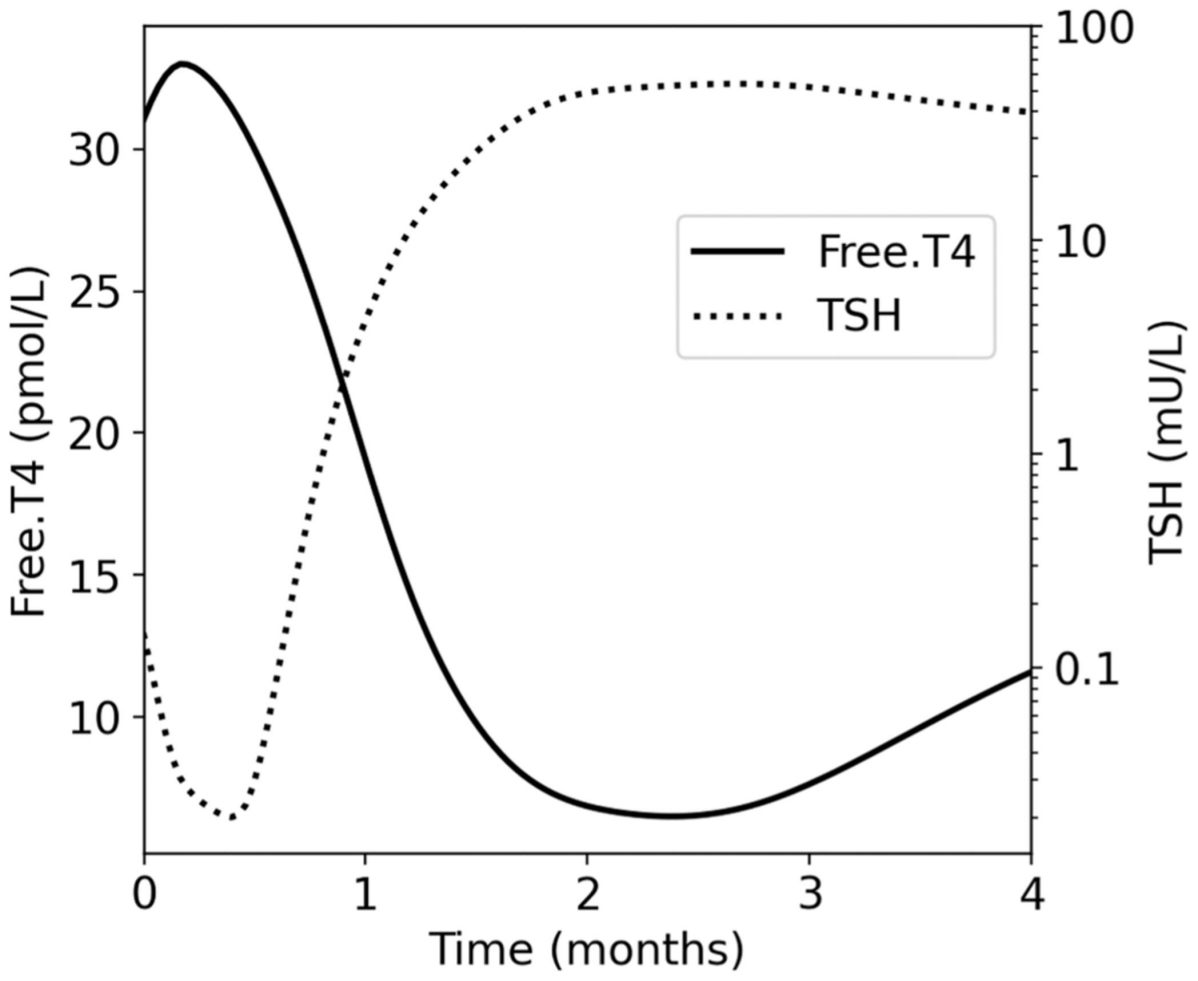
A plot showing median fT4 and TSH among patients who progressed from overt hyperthyroidism to overt hypothyroidism.

Of patients with overt hypothyroidism, 35.2% had no detected preceding hyperthyroidism. Most of those had normal thyroid function measured within 3 weeks of overt hypothyroidism onset (supplementary figure 3), suggesting hyperthyroidism did not occur or was short-lived. Fifty-seven patients with at least six weekly thyroid function measurements developed overt hyperthyroidism followed by overt hypothyroidism. The median time from overt hyperthyroidism to overt hypothyroidism among those patients was 1.6 months, while the first and ninth deciles, respectively, were 1.1 and 2.5 months. Median fT4 and TSH concentrations in these patients from overt hyperthyroidism onset until overt hypothyroidism are shown in Figure 2.

### 3.4 Baseline factors and thyroid dysfunction

**Fig. 3:**
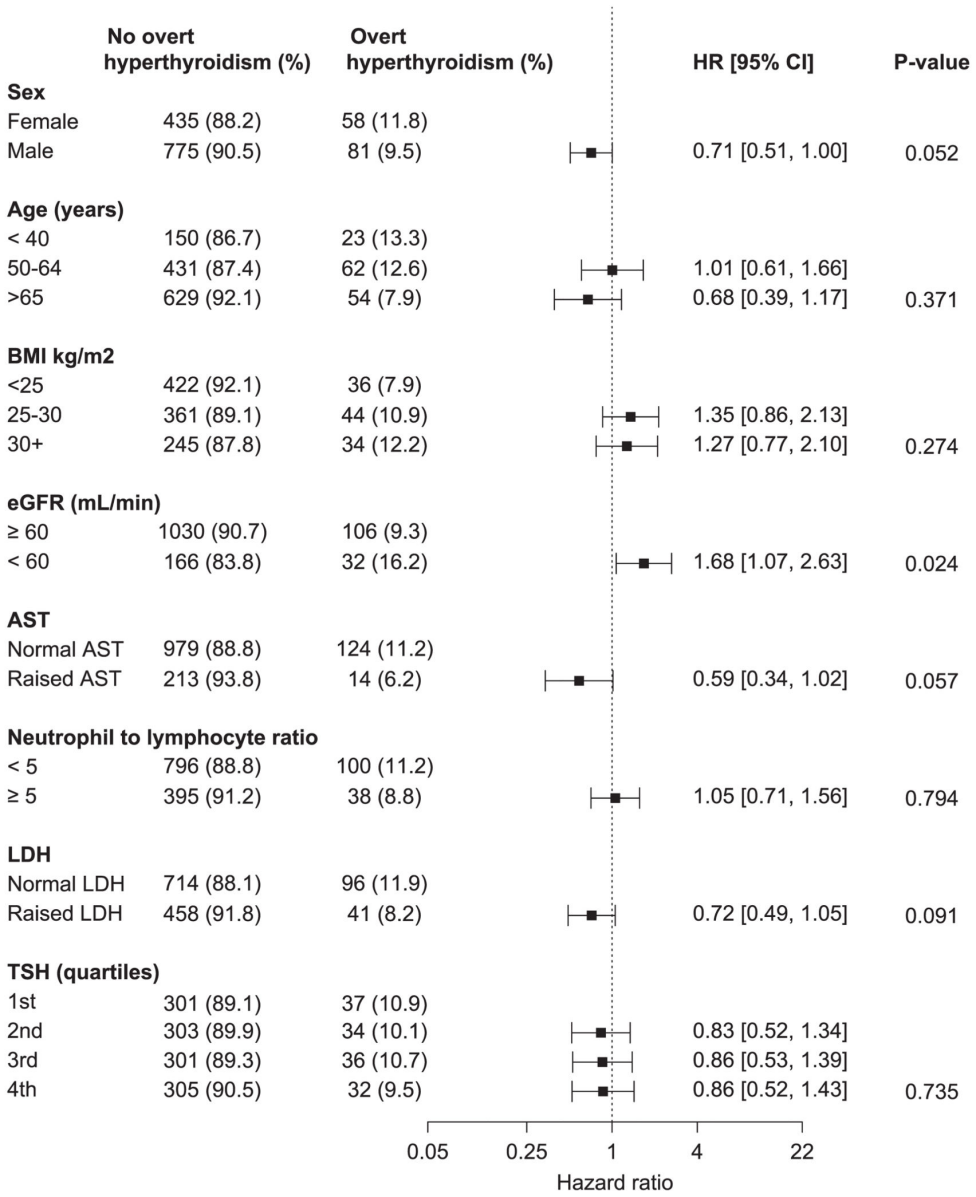
A forest plot showing HRs for associations between baseline factors and overt hyperthyroidism.

**Fig. 4:**
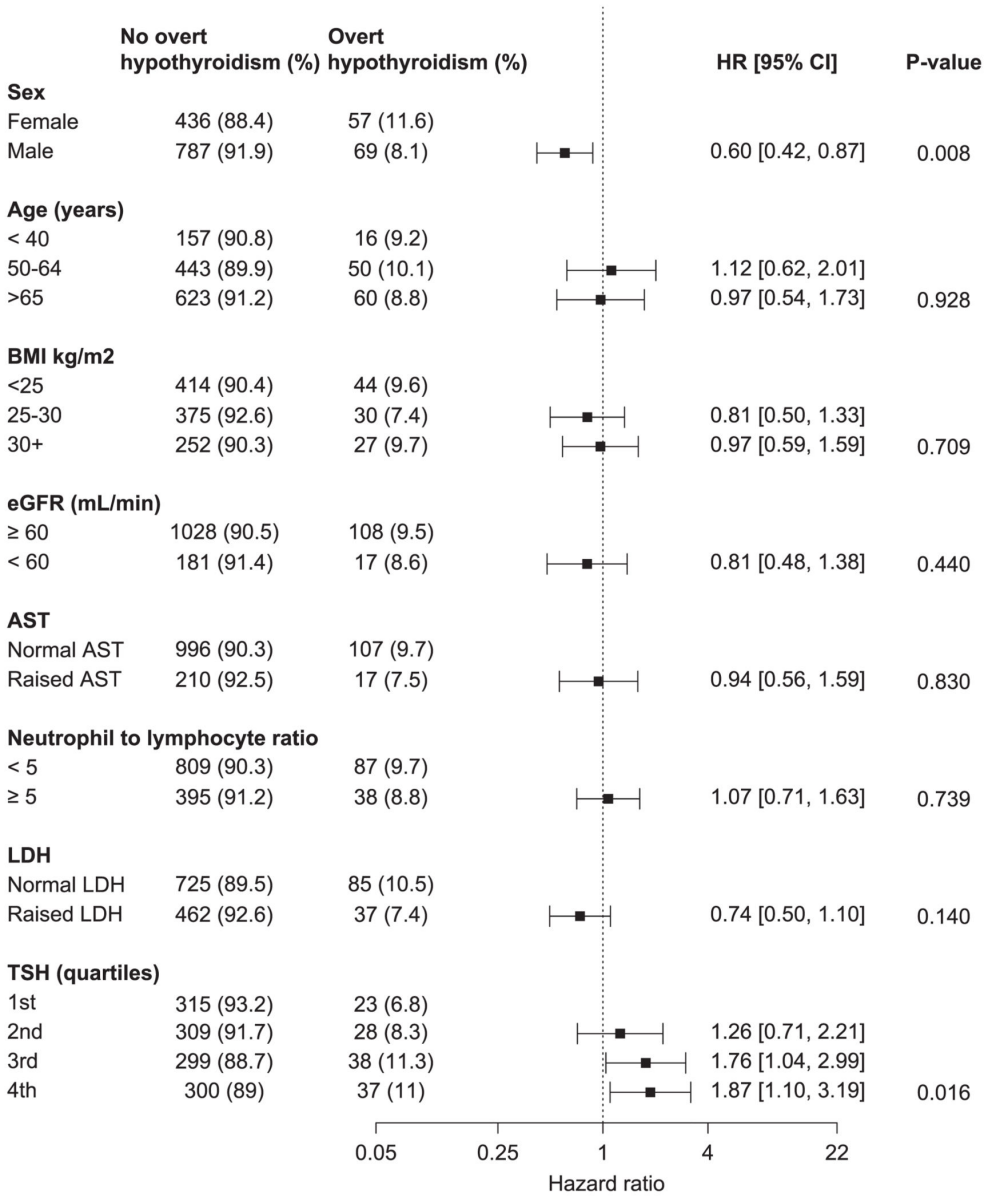
A forest plot showing HRs for associations between baseline factors and overt hypothyroidism.

Cumulative incidence curves for overt hyperthyroidism and overt hypothyroidism according to baseline covariates are shown in supplementary figures 4 to 25. Cumulative incidences of overt hypothyroidism and overt hyperthyroidism at 3, 6 and 12 months are shown in supplementary table 3. Most overt hyperthyroidism occurred by 3 months and overt hypothyroidism by 6 months. HRs for associations of baseline covariates with cumulative incidences of overt hyperthyroidism and hypothyroidism, respectively, are shown in Figures 3 and 4. eGFR < 60 mL/min/1.73 m^2^ (HR=1.68, 95% CI 1.07–2.63) was associated with overt hyperthyroidism. Sex (HR=0.60, 95% CI 0.42–0.87) and TSH (4th vs. 1st quartiles HR=1.87, 1.10–3.19) were associated with overt hypothyroidism. In sensitivity analyses investigating cumulative incidences of overt hyperthyroidism/hypothyroidism in the first six months among those who did not discontinue treatment, the aforementioned associations remained similar in direction but were not significant (supplementary table 4), possibly due to lower power from fewer cases. HRs for associations between treatment indication and thyroid dysfunction are shown in supplementary table 5.

### 3.5 Thyroid dysfunction and overall survival

**Fig. 5:**
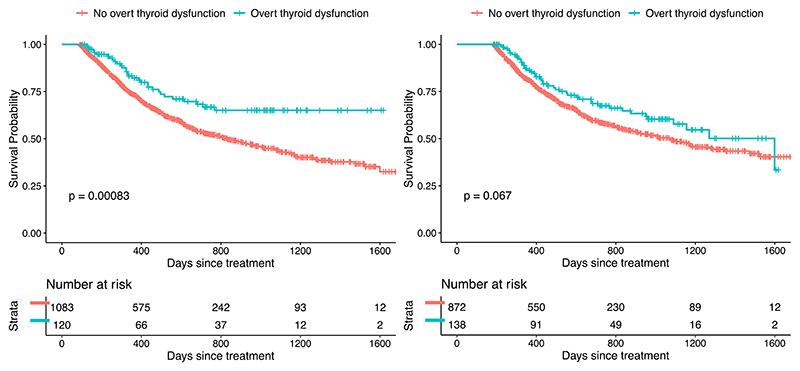
Kaplan-Meier plots of overall survival for overt thyroid dysfunction vs. normal thyroid function in the first 3 months (left) and 6 months (right) after treatment initiation.

During a median follow-up of 22.2 months (range 0.4–55.8 months) there were 562 OS events (41.6%). The 3-month and 6-month landmark analyses, respectively, included 1203 and 1010 patients, who had more favourable prognostic characteristics (supplementary table 6). Landmarked survival curves are shown in Figure 5 (overt dysfunction) and supplementary figures 26–28 (other types). Overt thyroid dysfunction was strongly associated with OS in the Cox analyses (HR=0.65, 0.50–0.85) but not in the time-dependent Cox analyses (HR=0.79, 0.60–1.03), 3-month landmark (HR=0.74, 0.51–1.07) or 6-month landmark (HR=0.91, 0.66–1.24). A similar pattern was seen for overt hyperthyroidism (supplementary table 7). Although there was an association between overt hypothyroidism and OS in the 3-month landmark analysis (HR=0.40, 0.17–0.98), this was not present in the 6-month landmark or time-dependent Cox analyses and so may have been influenced by exposure misclassification. In the Cox analysis, HRs for associations of subclinical hypothyroidism, subclinical hyperthyroidism, overt hypothyroidism (de novo) and overt hyperthyroidism with OS, respectively were 0.73 (0.56–0.94), 0.81 (0.64–1.01), 0.53 (0.34–0.82) and 0.62 (0.44–0.87) (supplementary table 8). The only significant association in the landmark analyses was for de novo overt hypothyroidism at 3 months (HR=0.24, 0.06–0.99) but again this may have been influenced by exposure misclassification. There was no strong evidence of interactions with ICI (p = 0.269) or cancer type (p = 0.103). HRs for associations between treatment indication and OS are shown in supplementary table 9.

## 4 Discussion

Thyroid dysfunction is one of the most common toxicities in patients receiving ICIs and results from immune damage to the thyroid gland. The response to this damage can result in different scenarios which can be both dynamic and unpredictable in outcome. We investigated thyroid dysfunction among 1349 patients treated with ICIs. Subclinical hyperthyroidism (18.8%) and subclinical hypothyroidism (16.2%) were the most common manifestations of thyroid damage. Overt hyperthyroidism and overt hypothyroidism occurred in 10.3% and 9.3%, respectively (22.3% and 14.0% with combination ICIs). Secondary dysfunction was rare (0.5%). The clinical course ranged from transient subclinical dysfunction to rapid progression (<2 months) from overt hyperthyroidism, with very high fT4s, to overt hypothyroidism. In approximately one third of cases, overt hypothyroidism occurred without preceding hyperthyroidism. The proportions of overt thyroid dysfunction seen with combination ipilimumab/nivolumab were similar in renal cancer and melanoma despite the ‘flipped’ ICI doses in the respective protocols. In melanoma, adjuvant treatment caused more frequent and aggressive dysfunction. Baseline eGFR < 60 mL/min/1.73 m^2^ was associated with overt hyperthyroidism, while sex and TSH were associated with overt hypothyroidism. Thyroid dysfunction was not strongly associated with OS when using methods accounting for immortal time bias.

Clinical trials have generally reported lower rates of thyroid dysfunction than the present study (e.g. 14.3% and 17% with hypothyroidism in KEYNOTE-054 [2] and Checkmate-067 [16]). Trials may not capture all subclinical cases because the Common Terminology Criteria for Adverse Events [23] defines hyperthyroidism/hypothyroidism by ‘thyroid hormone’ levels and not TSH. The results of this study are similar to other real-world studies, which report dysfunction rates of 17% to 62.0% and onset times of 31–63 days for hyperthyroidism and 98–105 days for hypothyroidism [10,17,18,20, 24].

Previous studies have been inconsistent regarding rates of thyroid recovery after ICI-induced dysfunction. A large study involving melanoma patients reported that most patients with overt hyperthyroidism recovered [19], while another study found that 100% with hyperthyroidism (denoted by raised fT4) eventually required thyroxine therapy [20]. The potential of avoiding progression to permanent hypothyroidism may merit further investigation, particularly in adjuvant patients who had more frequent and aggressive dysfunction in this study. Such patients, who may have a more intact immune system [25], are at higher risk of longer-term effects of thyroid disorders on quality of life and mortality [13–15]. However, consideration needs also to be given to the potential adverse impact of immunosuppressive therapy on treatment effectiveness.

Few studies have investigated the velocity of changes in fT4 and TSH in thyrotoxicosis and times until subsequent hypothyroidism (where recovery does not occur). In non-ICI thyroiditis, fT4 remains elevated and falls below normal after ~6 months [26]. In the present study, some patients progressed from overt hyperthyroidism to overt hypothyroidism in < 6 weeks. Rapidly progressive ICI-induced thyroid dysfunction and rapid onset de novo overt hypothyroidism have implications for monitoring.

Previous studies have reported associations of female sex, younger age, BMI and TSH with ICI-induced thyroid dysfunction [10,18,19,27], but not eGFR. The association with eGFR in this study may reflect the complex relationship between chronic kidney disease (CKD) and thyroid function [28]. Alternatively, it may have been due to chance or residual confounding by more aggressive treatments and lower eGFR in renal cancer. Associations of thyroid dysfunction with PFS [10] and OS [11] have been reported previously. This study replicated the association with OS using similar methods, but the association was not significant when immortal time bias was accounted for and so may be less important than previously thought.

The present study had some important limitations. Thyroid replacement therapy may have led to missed cases or misclassification of iatrogenic thyroid dysfunction. Thyroid dysfunction due to ‘non-thyroidal illnesses’ [29] may have been misclassified as isolated hypothyroxinaemia or secondary overt hypothyroidism, though patients were generally low-risk for this from baseline characteristics. Secondary hypothyroidism (rare with PD-L1/PD-1 ICIs [5,30]) may have been misclassified as subclinical hypothyroidism (or hypothyroxinaemia) given their biochemical similarities. The prevalence of antibody positive thyroid dysfunction was not estimated. The baseline variable-thyroid dysfunction associations may have been biased by changing testing likelihood (e.g. with discontinuation), though they remained similar in sensitivity analysis including only patients who did not discontinue treatment. In addition, the associations may not uniformly apply across cancer types and ICIs since we did not test for interactions due to small numbers of events in some categories. The landmark analyses may have been affected by exclusions (reducing power and resulting in patients with better prognoses) and exposure misclassification, particularly of overt hypothyroidism at the 3-month landmark. Finally, low statistical power from relatively few OS events in the follow-up time and heterogeneity in our cohort may have limited the ability to detect small effects in the survival analyses.

In summary, this study found high rates of ICI-induced thyroid dysfunction. Clinical courses included temporary dysfunction, rapidly progressive thyrotoxicosis and de novo overt hypothyroidism. Reduced renal function may be a risk factor for overt hyperthyroidism. Thyroid dysfunction did not consistently predict longer survival. Further studies are merited given the increasing use of ICIs, particularly for lower stage cancers, and the potential for thyroid dysfunction to affect longer-term health outcomes.

## Supplementary Material

**Appendix A. Supporting information**

Supplementary data associated with this article can be found in the online version at doi:10.1016/j.ejca.2024.113949.

Supplementary Material

## Figures and Tables

**Table 1:** Baseline characteristics of included patients according to treatment and diagnosis.

	All	Treatment									Diagnosis					
		Pembro	Nivolumab	Ipi + nivo	ChemoIO	Atezo	Avelumab	Durvalumab	Other		Lung	Melanoma	Renal	H, N & F	LGI	Other
**Sex**																
Female	493 (36.5)	154 (37.7)	69 (30.0)	69 (32.1)	95 (49.0)	57 (42.2)	17 (27.9)	22 (36.1)	10 (22.7)		160 (42.9)	133 (40.1)	39 (20.4)	19 (18.1)	33 (42.3)	109 (40.4)
Male	856 (63.5)	255 (62.3)	161 (70.0)	146 (67.9)	99 (51.0)	78 (57.8)	44 (72.1)	39 (63.9)	34 (77.3)		213 (57.1)	199 (59.9)	152 (79.6)	86 (81.9)	45 (57.7)	161 (59.6)
**Age (years)**																
< 50	173 (12.8)	36 (8.8)	46 (20.0)	40 (18.6)	23 (11.8)	12 (8.9)	2 (3.3)	6 (9.8)	8 (18.2)		12 (3.2)	56 (16.8)	22 (11.5)	8 (7.6)	20 (25.6)	55 (20.4)
50-64	494 (36.6)	137 (33.4)	83 (36.1)	98 (45.6)	79 (40.5)	40 (29.6)	17 (27.9)	29 (47.5)	11 (25.0)		134 (35.8)	102 (30.6)	86 (45.0)	54 (51.4)	27 (34.6)	91 (33.7)
≥65	684 (50.6)	237 (57.8)	101 (43.9)	77 (35.8)	93 (47.7)	83 (61.5)	42 (68.9)	26 (42.6)	25 (56.8)		228 (61.0)	175 (52.6)	83 (43.5)	43 (41.0)	31 (39.7)	124 (45.9)
**ECOG PS**																
0	773 (57.3)	228 (55.7)	127 (55.2)	158 (73.5)	112 (57.7)	60 (44.4)	36 (59.0)	34 (55.7)	18 (40.9)		203 (54.4)	221 (66.6)	123 (64.4)	52 (49.5)	52 (66.7)	122 (45.2)
1	360 (26.7)	119 (29.1)	59 (25.7)	37 (17.2)	56 (28.9)	39 (28.9)	19 (31.1)	19 (31.1)	12 (27.3)		122 (32.7)	72 (21.7)	44 (23.0)	46 (43.8)	14 (17.9)	62 (23.0)
≥2	43 (3.2)	19 (4.6)	7 (3.0)	3 (1.4)	4 (2.1)	7 (5.2)	2 (3.3)	0 (0.0)	1 (2.3)		11 (2.9)	13 (3.9)	5 (2.6)	2 (1.9)	4 (5.1)	8 (3.0)
**BMI kg/m2**																
< 18.5	458 (33.9)	141 (34.4)	100 (43.5)	55 (25.6)	75 (38.5)	50 (37.0)	0 (0.0)	24 (39.3)	13 (29.5)		157 (42.0)	81 (24.3)	35 (18.3)	72 (68.6)	29 (37.2)	84 (31.1)
18.5-24.9	407 (30.1)	126 (30.7)	62 (27.0)	70 (32.6)	67 (34.4)	49 (36.3)	0 (0.0)	20 (32.8)	13 (29.5)		123 (32.9)	106 (31.8)	41 (21.5)	17 (16.2)	28 (35.9)	92 (34.1)
25-29.9	279 (20.7)	89 (21.7)	43 (18.7)	65 (30.2)	42 (21.5)	22 (16.3)	0 (0.0)	13 (21.3)	5 (11.4)		53 (14.2)	99 (29.7)	42 (22.0)	11 (10.5)	19 (24.4)	55 (20.4)
**eGFR (mL/min/1.73 m^2^)**																
≥ 60	1136 (84.2)	350 (85.6)	205 (89.1)	170 (79.1)	187 (96.4)	105 (77.8)	35 (57.4)	51 (83.6)	33 (75.0)		338 (90.6)	297 (89.5)	107 (56.0)	100 (95.2)	73 (93.6)	221 (81.9)
<60	198 (14.7)	55 (13.4)	22 (9.6)	42 (19.5)	5 (2.6)	30 (22.2)	25 (41.0)	9 (14.8)	10 (22.7)		32 (8.6)	32 (9.6)	83 (43.5)	3 (2.9)	5 (6.4)	43 (15.9)
**AST (U/L)**																
Normal	1103 (81.8)	344 (84.1)	181 (78.7)	175 (81.4)	143 (73.7)	106 (78.5)	57 (93.4)	58 (95.1)	39 (88.6)		316 (84.7)	279 (84.0)	168 (88.0)	85 (81.0)	55 (70.5)	200 (74.1)
Raised	227 (16.8)	57 (13.9)	46 (20.0)	37 (17.2)	49 (25.3)	29 (21.5)	3 (4.9)	2 (3.3)	4 (9.1)		52 (13.9)	49 (14.8)	22 (11.5)	17 (16.2)	23 (29.5)	64 (23.7)
**NLR**																
<5	896 (66.4)	292 (71.4)	136 (59.1)	163 (75.8)	118 (60.8)	79 (58.5)	47 (77.0)	38 (62.3)	23 (52.3)		199 (53.4)	277 (83.4)	156 (81.7)	42 (40.0)	48 (61.5)	174 (64.4)
≥5	433 (32.1)	113 (27.6)	91 (39.6)	47 (21.9)	72 (37.1)	56 (41.5)	12 (19.7)	22 (36.1)	20 (45.5)		170 (45.6)	50 (15.1)	34 (17.8)	63 (60.0)	28 (35.9)	88 (32.6)
**LDH**																
Normal	810 (60.0)	248 (60.6)	128 (55.7)	138 (64.2)	99 (51.0)	74 (54.8)	36 (59.0)	53 (86.9)	34 (48.5)		181 (77.3)	222 (66.9)	124 (64.9)	78 (74.3)	42 (53.8)	163 (60.4)
Raised	499 (37.0)	143 (35.0)	97 (42.2)	72 (33.5)	90 (46.4)	59 (43.7)	23 (37.7)	7 (11.5)	8 (18.2)		180 (48.3)	101 (30.4)	62 (32.5)	22 (21.0)	35 (44.9)	99 (36.7)

Abbreviations: niv (nivolumab), ipi (ipilimumab), atezo (atezolizumab), pembro (pembrolizumab), chemoIO (chemoimmunotherapy), BMI (body mass index), eGFR (estimated glomerular filtration rate), AST (aspartate aminotransferase), NLR (neutrophil lymphocyte ratio), H, N & F (head, neck and face), LGI (lower gastrointestinal), ECOG PS (Eastern Cooperative Oncology Group performance status)

**Table 2:** Proportions of patients that developed hyperthyroidism with median times to onset, maximum fT4 and median fT4.

Hyperthyroidism									
	N	Total (%)	Subclinical (%)	Isolated ↑T4 (%)	Overt (%)	Median onset (m)^†^	Max T4 (pmol/L)	Median T4 (pmol/L) ^†^	Median time to last TFT (months)
**All**	1349 * *	462 (34.2)	253 (18.8)	68 (5.0)	139 (10.3)	1.4	144.8	28.5	8.2
**Treatment**									
Pembrolizumab	409	119 (29.1)	73 (17.8)	12 (2.9)	34 (8.3)	1.6	85	28.3	8.6
Nivolumab	230	61 (26.5)	33 (14.3)	9 (3.9)	19 (8.3)	1.8	58.8	26.4	7.1
Ipilimumab + nivolumab	215 *	118 (54.9)	52 (24.2)	17 (7.9)	48 (22.3)	1.3	144.8	32	12.7
ChemoIO	194	67 (34.5)	43 (22.2)	10 (5.2)	14 (7.2)	2	29.9	25.4	5.6
Atezolizumab	135	34 (25.2)	23 (17.0)	7 (5.2)	4 (3.0)	5.6	49.4	32.65	5.9
Other / unknown	166 *	63 (38.0)	29 (17.5)	13 (7.8)	20 (12.0)	0.9	48.5	33.4	10.5
**Diagnosis**									
Lung	373	115 (30.8)	77 (20.6)	15 (4.0)	23 (6.2)	1.5	79.9	25.5	6.7
Melanoma	332	128 (38.6)	70 (21.1)	14 (4.2)	44 (13.3)	1.3	87.9	32.55	11
Renal	191 *	97 (50.8)	36 (18.8)	18 (9.4)	42 (22.0)	1.3	144.8	32	15.3
H, N and F	105	23 (21.9)	14 (13.3)	2 (1.9)	7 (6.7)	1.8	40.4	24.6	4.1
LGI	78	22 (28.2)	12 (15.4)	4 (5.1)	6 (7.7)	1.5	85	25.1	3.8
Other	270 *	77 (28.5)	44 (16.3)	15 (5.6)	17 (6.3)	2.6	78	28.2	6
**Treatment intent (melanoma)**									
Adjuvant (pem=98 niv=26)	124	44 (35.5)	25 (20.2)	4 (3.2)	15 (12.1)	1.7	68.8	30.6	11.1
Metastatic (pem=71 niv=22)	93	28 (30.1)	19 (20.4)	2 (2.2)	7 (7.5)	2	40.3	27.5	9.8
**Combination ipilimumab + nivolumab**									
Melanoma (ipi 3 mg/kg + niv 1 mg/kg)	103	52 (50.5)	24 (23.3)	7 (6.8)	21 (20.4)	1.2	87.9	38.4	9.2
Renal (ipi 3 mg/kg + niv 1 mg/kg)	101 *	61 (60.4)	25 (24.8)	10 (9.9)	25 (24.8)	1.6	144.8	28.5	17.7

Abbreviations: chemoIO (chemoimmunotherapy), TSH (thyroid stimulating hormone), H, N & F (head, neck and face), LGI (lower gastrointestinal), m (months), pem (pembrolizumab), niv (nivolumab), ipi (ipilimumab), TFT (thyroid function test).

* includes 2 patients with secondary hyperthyroidism,

* * includes 1 patient with secondary hyperthyroidism,

† overt hyperthyroidism

**Table 3:** Proportions of patients that developed hypothyroidism with median times to onset.

	Hypothyroidism						
	N	Total (%)	Subclinical (%)	Isolated ↓T4 (%)	Overt (%)	Secondary (%)	Median onset (m) ^*^
**All**	1349	381 (28.2)	219 (16.2)	29 (2.1)	126 (9.3)	7 (0.5)	3.4
**Treatment**							
Pembrolizumab	409	109 (26.7)	58 (14.2)	2 (0.5)	48 (11.7)	1 (0.2)	3.4
Nivolumab	230	77 (33.5)	51 (22.2)	8 (3.5)	17 (7.4)	1 (0.4)	3.2
Ipilimumab + nivolumab	215	90 (41.9)	42 (19.5)	13 (6.0)	30 (14.0)	5 (2.3)	3.4
ChemolO	194	30 (15.5)	19 (9.8)	3 (1.5)	8 (4.1)	0 (0.0)	4
Atezolizumab	135	23 (17.0)	17 (12.6)	1 (0.7)	5 (3.7)	0 (0.0)	4.1
Other	166	52 (31.3)	32 (19.3)	2 (1.2)	18 (10.8)	0 (0.0)	3.5
**Diagnosis**							
Lung	373	60 (16.1)	28 (7.5)	3 (0.8)	29 (7.8)	0 (0.0)	4.2
Melanoma	332	91 (27.4)	37 (11.1)	11 (3.3)	42 (12.7)	1 (0.3)	3.2
Renal	191	105 (55.0)	69 (36.1)	6 (3.1)	26 (13.6)	4 (2.1)	3.5
H, N and F	105	38 (36.2)	23 (21.9)	3 (2.9)	12 (11.4)	0 (0.0)	3.3
LGI	78	27 (34.6)	16 (20.5)	2 (2.6)	7 (9.0)	2 (2.6)	2.7
Other / unknown	270	60 (22.2)	46 (17.0)	4 (1.5)	10 (3.7)	0 (0.0)	4.6
**Treatment intent (melanoma)**							
Adjuvant (pem=98 niv=26)	124	32 (25.8)	14 (11.3)	0 (0.0)	18 (14.5)	0 (0.0)	3.2
Metastatic (pem=71 niv=22)	93	19 (20.4)	9 (9.7)	1 (1.1)	9 (9.7)	0 (0.0)	4.4
**Combination ipilimumab + nivolumab**							
Melanoma (ipi 3 mg/kg + niv 1 mg/kg)	103	36 (35.0)	11 (10.7)	9 (8.7)	15 (14.6)	1 (1.0)	2.7
Renal (ipi 3 mg/kg + niv 1 mg/kg)	101	51 (50.5)	29 (28.7)	4 (4.0)	14 (13.9)	4 (4.0)	4.3

Abbreviations: I+N (ipilimumab and nivolumab), chemoIO (chemoimmunotherapy), TSH (thyroid stimulating hormone), H, N & F (head, neck and face), LGI (lower gastrointestinal), m (months), pem (pembrolizumab), niv (nivolumab), ipi (ipilimumab), TFT (thyroid function test).

* overt hypothyroidism
