# Supplementary material for: Thyroid dysfunction after immune checkpoint inhibitors in a single-centre UK pan-cancer cohort: A retrospective study
Source: Eur J Cancer. Author manuscript; Available in PMC 2026 Jan 16. (PMC7618629; doi:10.1016/j.ejca.2024.113949)
Supplement: Supplementary Material [file EMS212056-supplement-Supplementary_Material.pdf]

Thyroid dysfunction after immune checkpoint inhibitors in a single-centre UK pan-cancer cohort: a retrospective study – supplementary information

Oliver John Kennedy[a,b,*], Nadia Ali[a], Rebecca Lee[a,b], Phillip Monaghan[b,c], Safwaan Adam[a,b], Tim Cooksley[a], Paul Lorigan[a,b]

a. The Christie NHS Foundation Trust, Manchester, M20 4BX, UK

b. Division of Cancer Sciences, The University of Manchester, Manchester, M13 9PL, UK

c. The Christie Pathology Partnership, Manchester, M20 4BX, UK

*corresponding author

Short title: Immune checkpoint inhibitors and thyroid function

Keywords: Immune checkpoint inhibitors, immunotherapy, thyroiditis, hyperthyroidism, hypothyroidism, overall survival

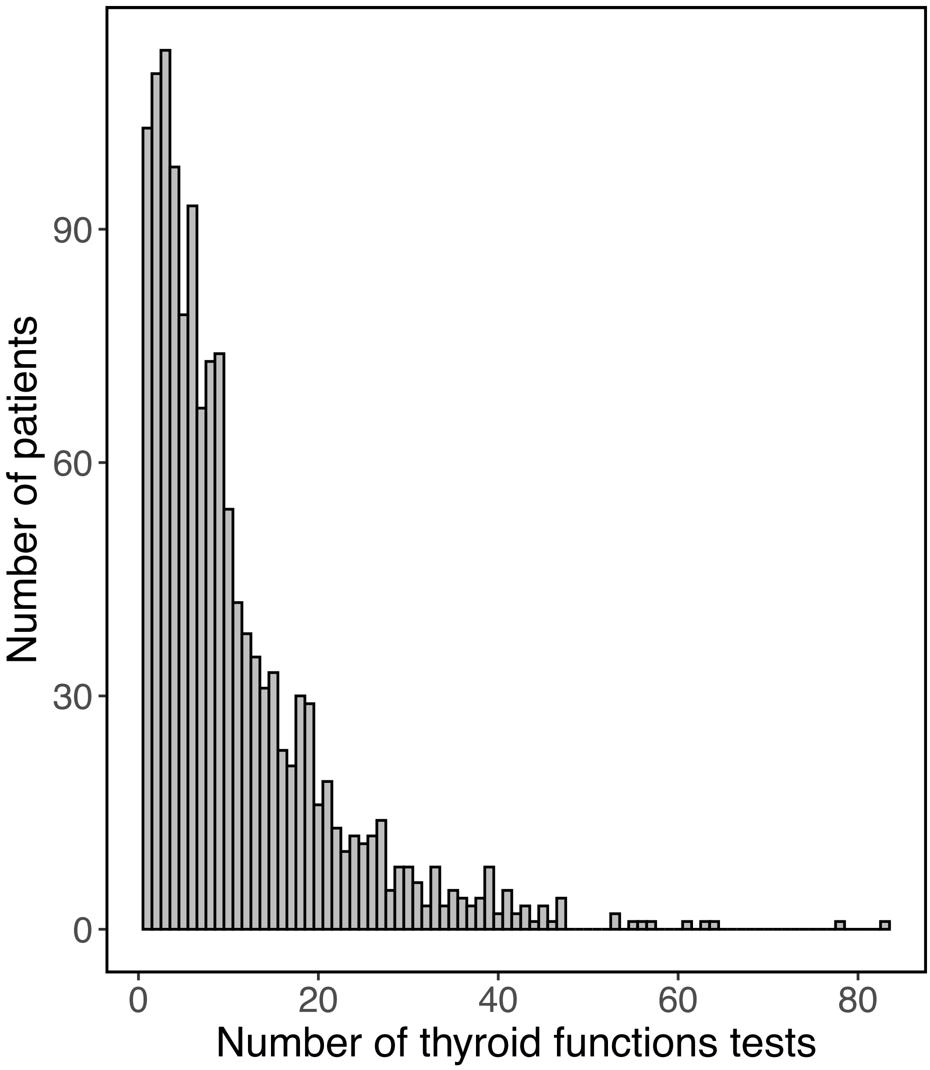

Supplementary figure 1. Histogram showing the number of thyroid function tests for each patient during follow-up.

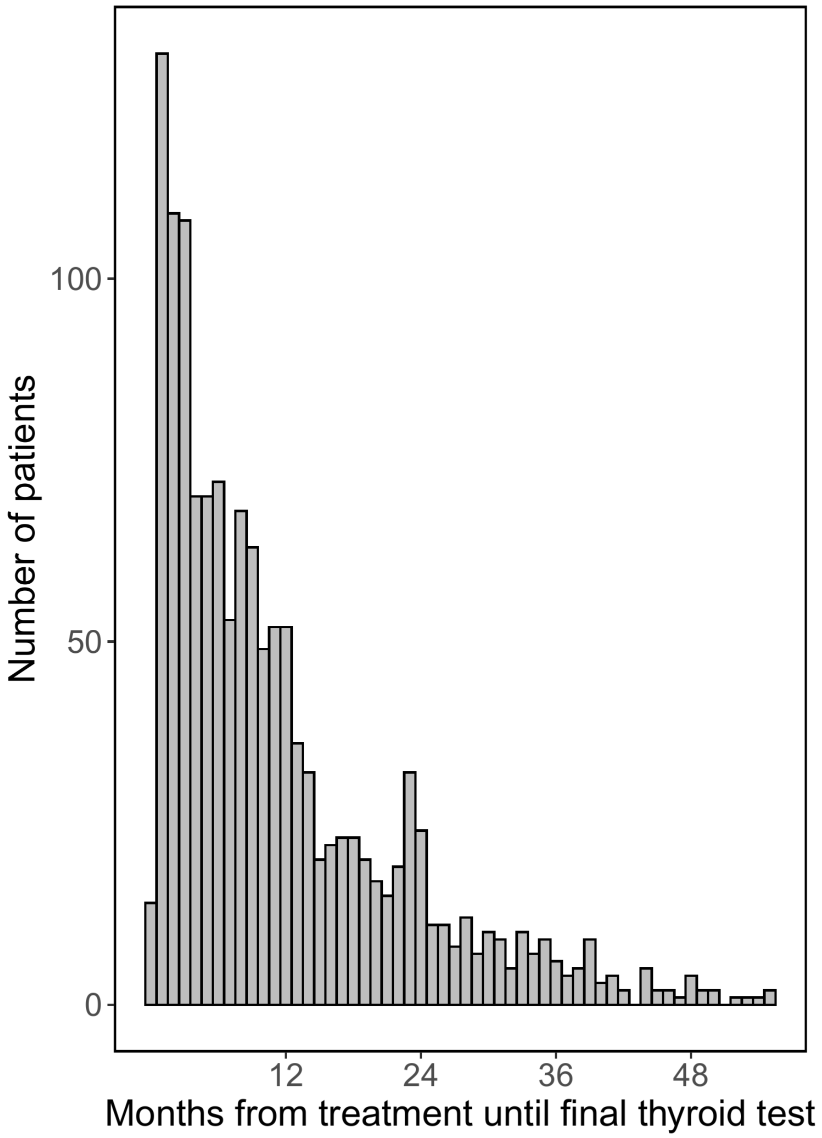

Supplementary figure 2. Histogram showing the time from treatment until the final thyroid function test for each patient.

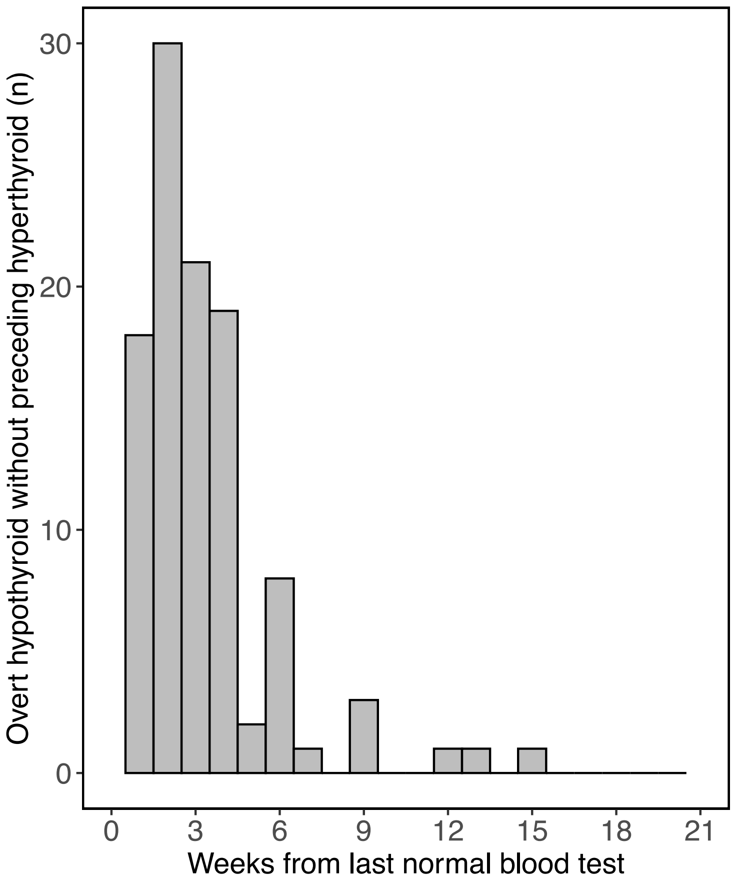

Supplementary figure 3. Histogram showing weeks since the previous normal thyroid function blood test in patients with incident hypothyroidism without detected preceding hyperthyroidism.

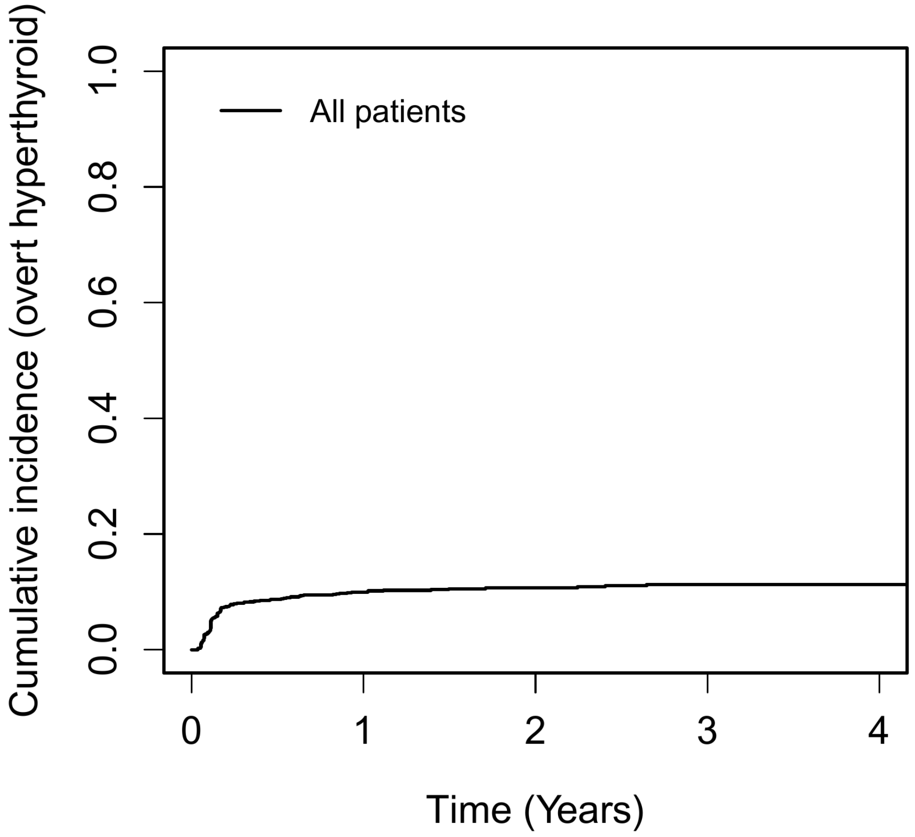

Supplementary figure 4. Cumulative incidence of overt hyperthyroidism among all patients.

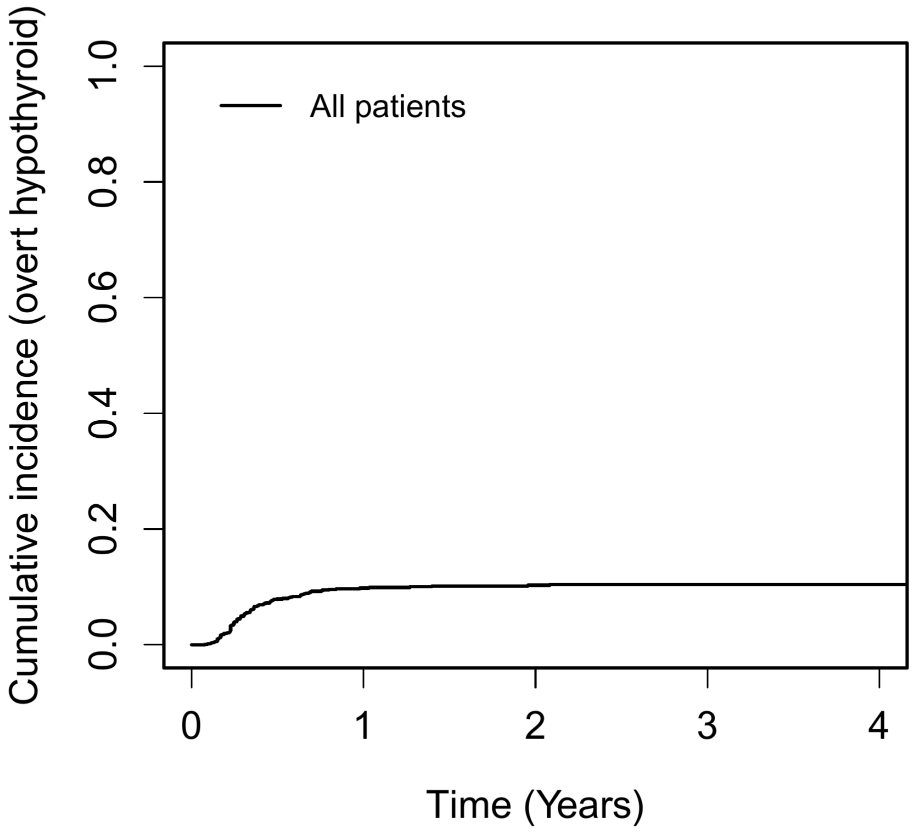

Supplementary figure 5. Cumulative incidence of overt hypothyroidism among all patients.

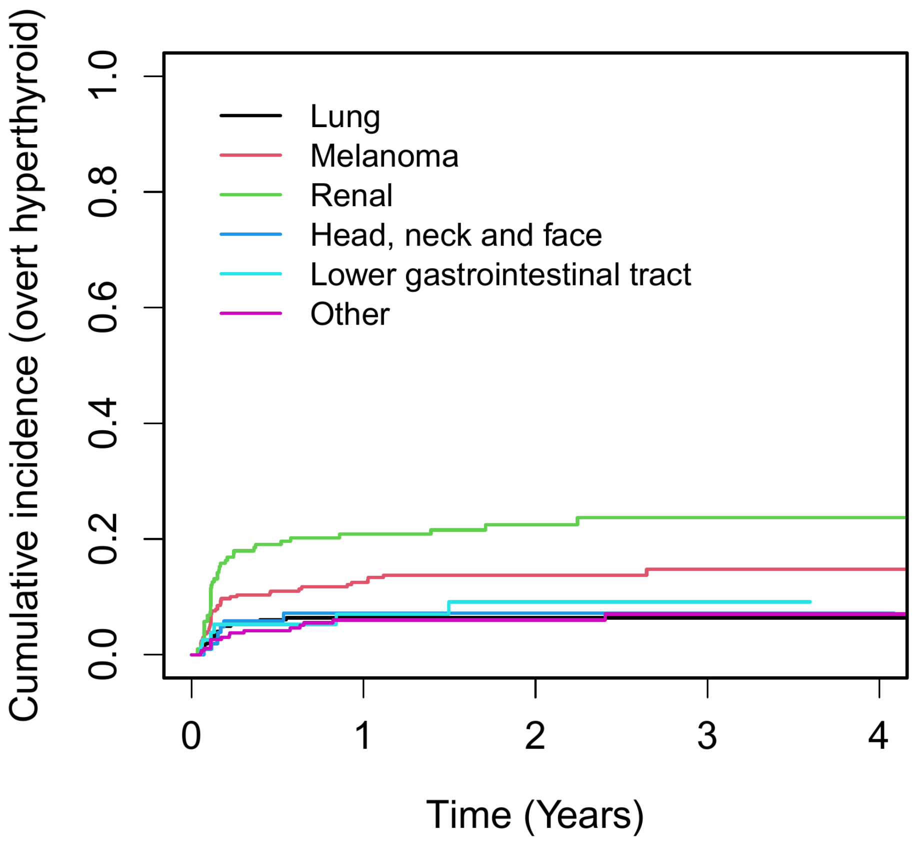

Supplementary figure 6. Cumulative incidence of overt hyperthyroidism according to diagnosis.

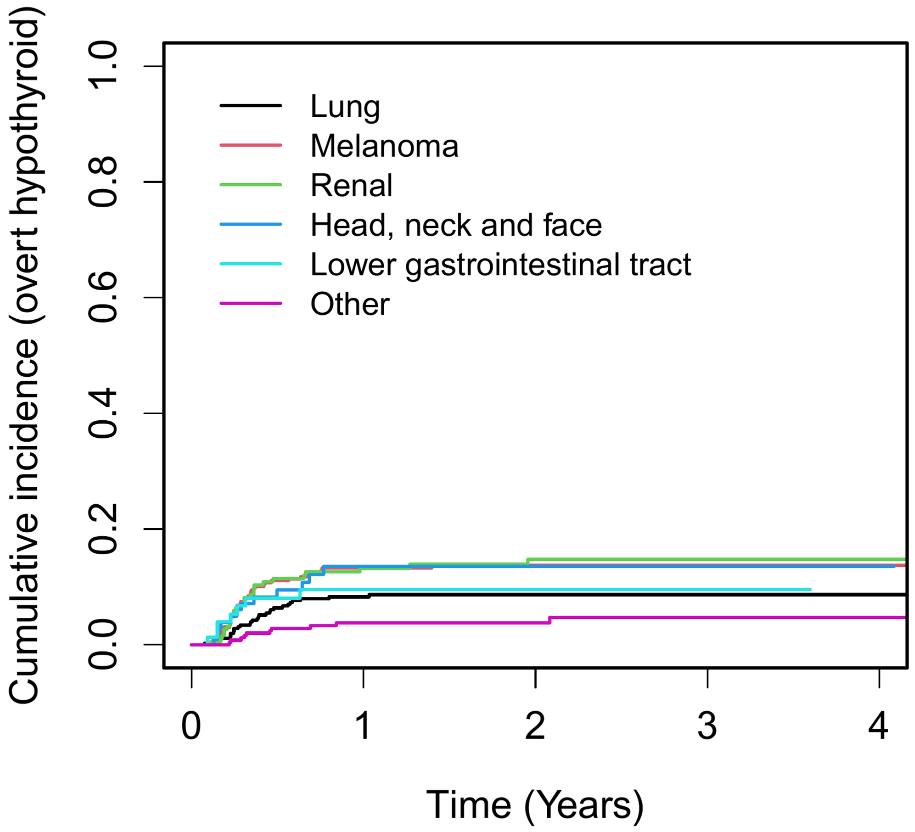

Supplementary figure 7. Cumulative incidence of overt hypothyroidism according to diagnosis.

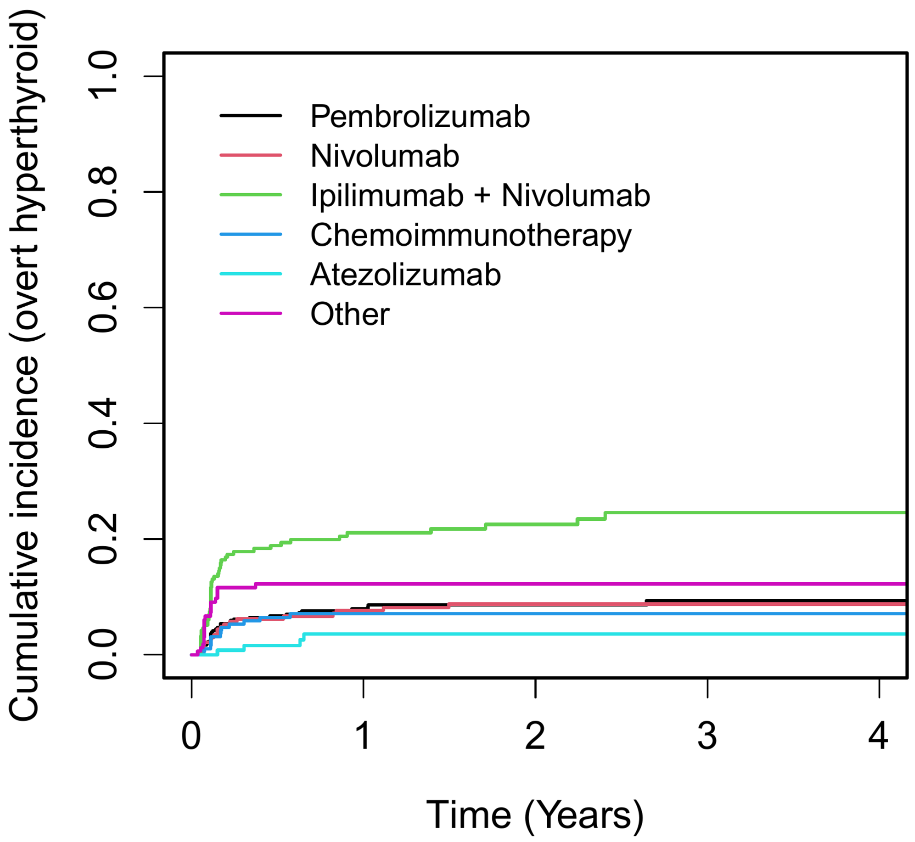

Supplementary figure 8. Cumulative incidence of overt hyperthyroidism according to immune checkpoint inhibitor.

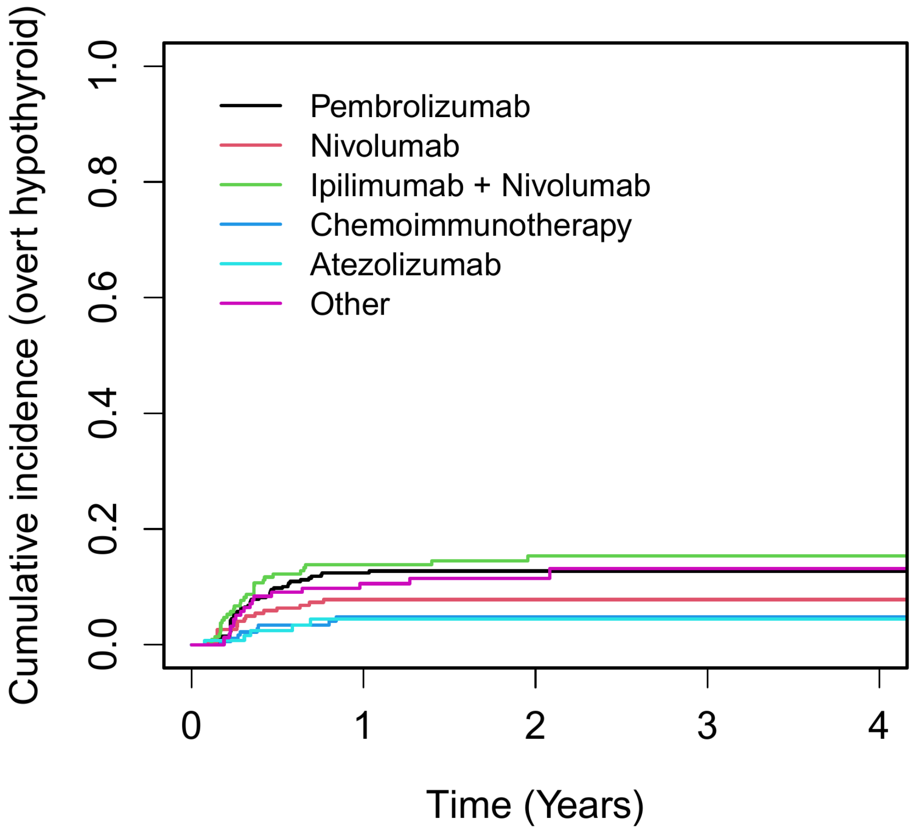

Supplementary figure 9. Cumulative incidence of overt hypothyroidism according to immune checkpoint inhibitor.

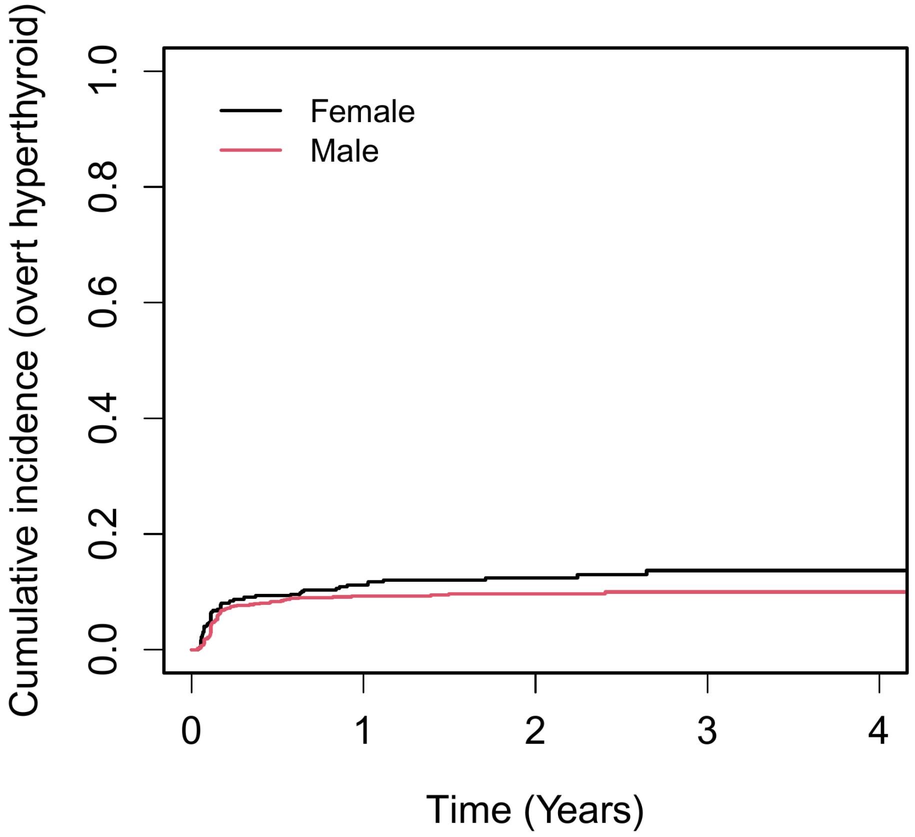

Supplementary figure 10. Cumulative incidence of overt hyperthyroidism according to sex.

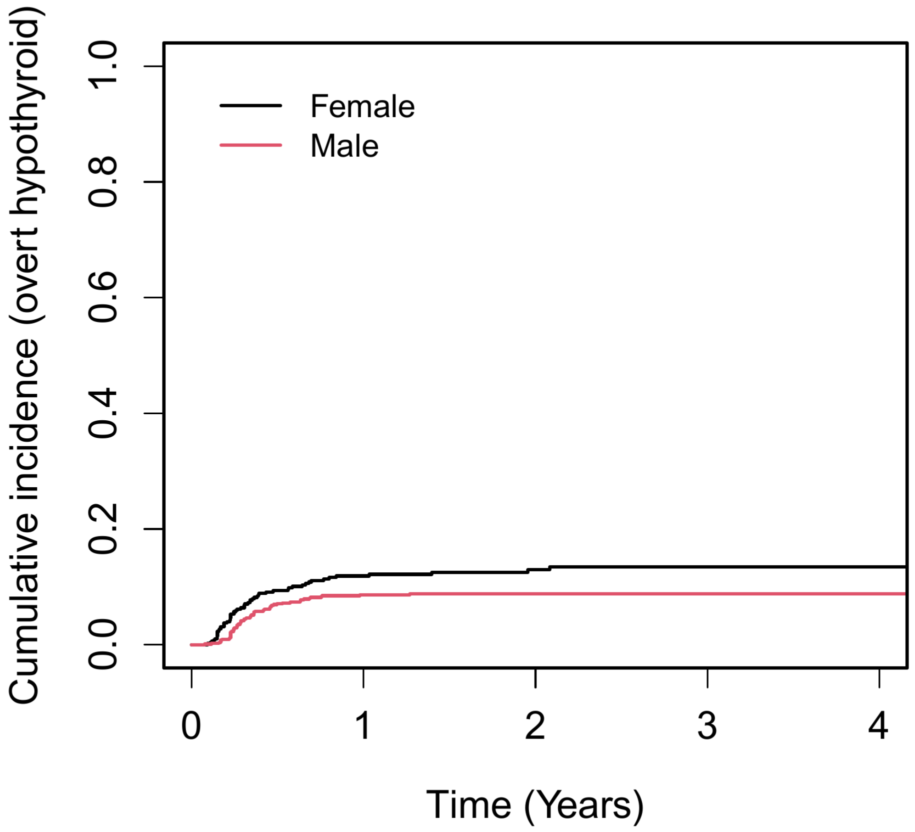

Supplementary figure 11. Cumulative incidence of overt hypothyroidism according to sex.

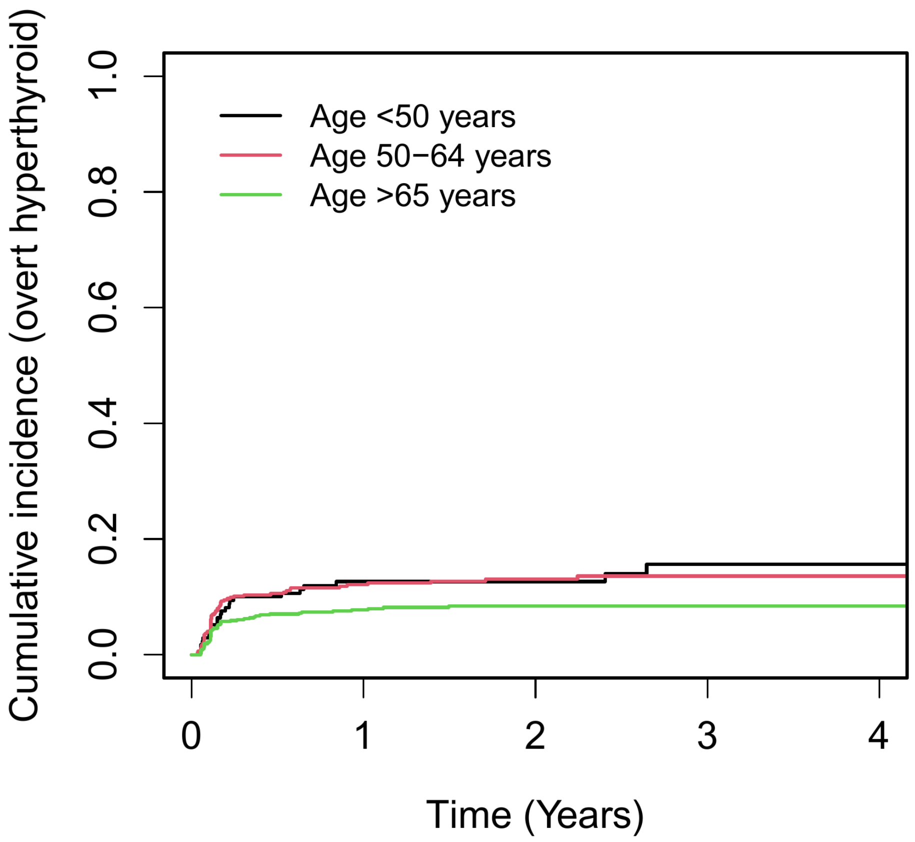

Supplementary figure 12. Cumulative incidence of overt hyperthyroidism according to age at baseline.

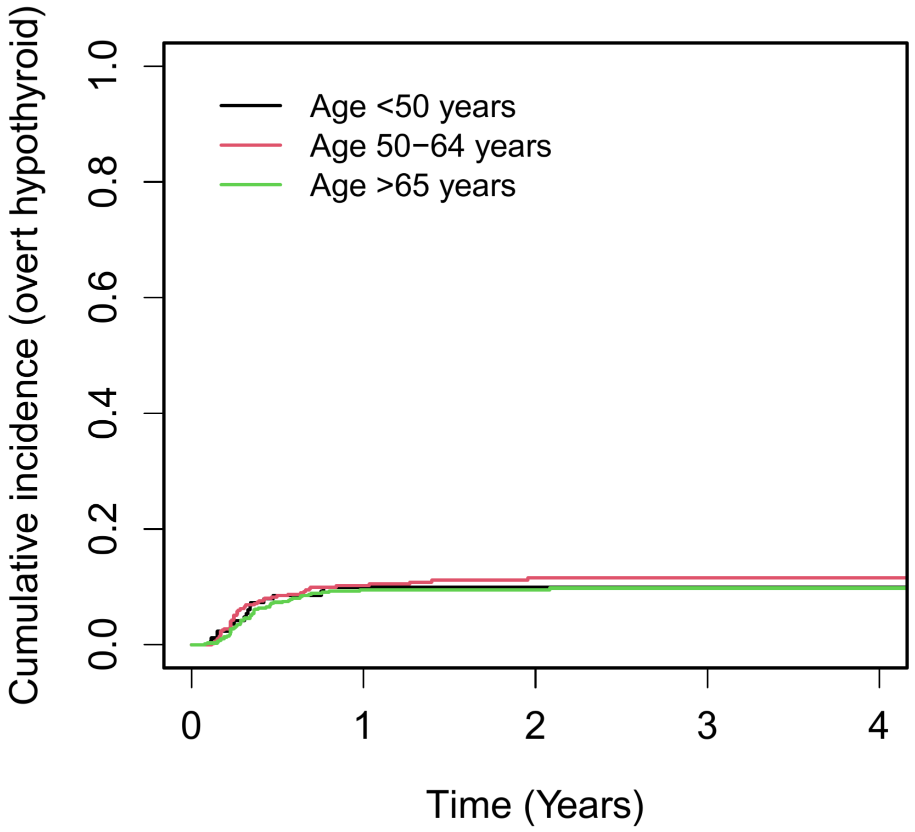

Supplementary figure 13. Cumulative incidence of overt hypothyroidism according to age at baseline.

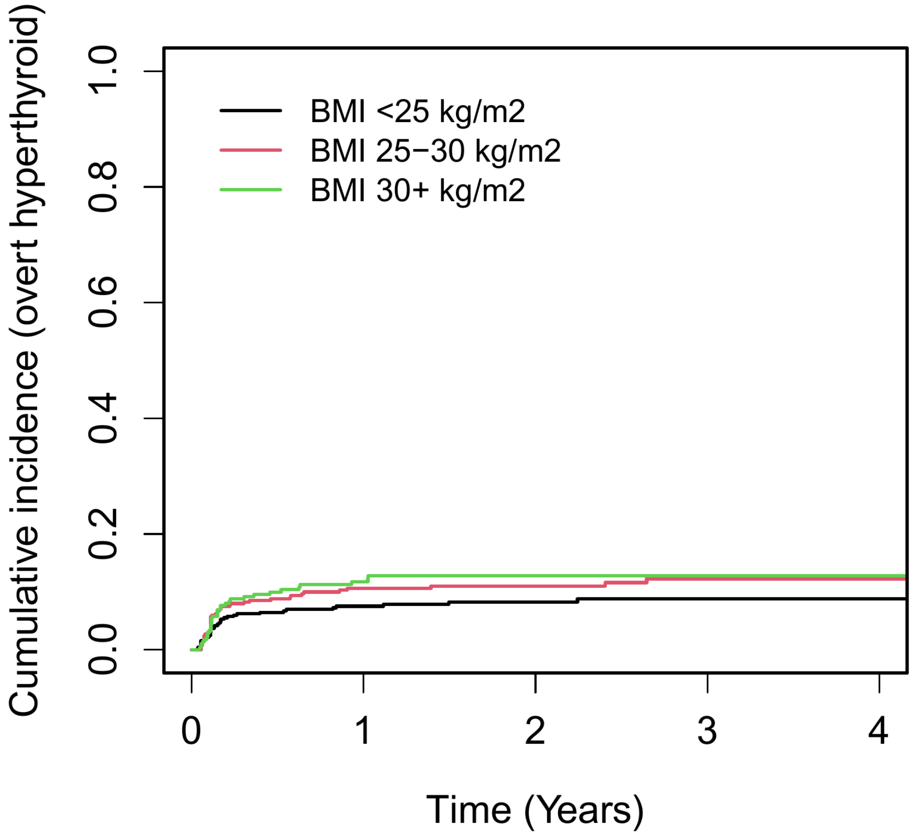

Supplementary figure 14. Cumulative incidence of overt hyperthyroidism according to baseline body mass index.

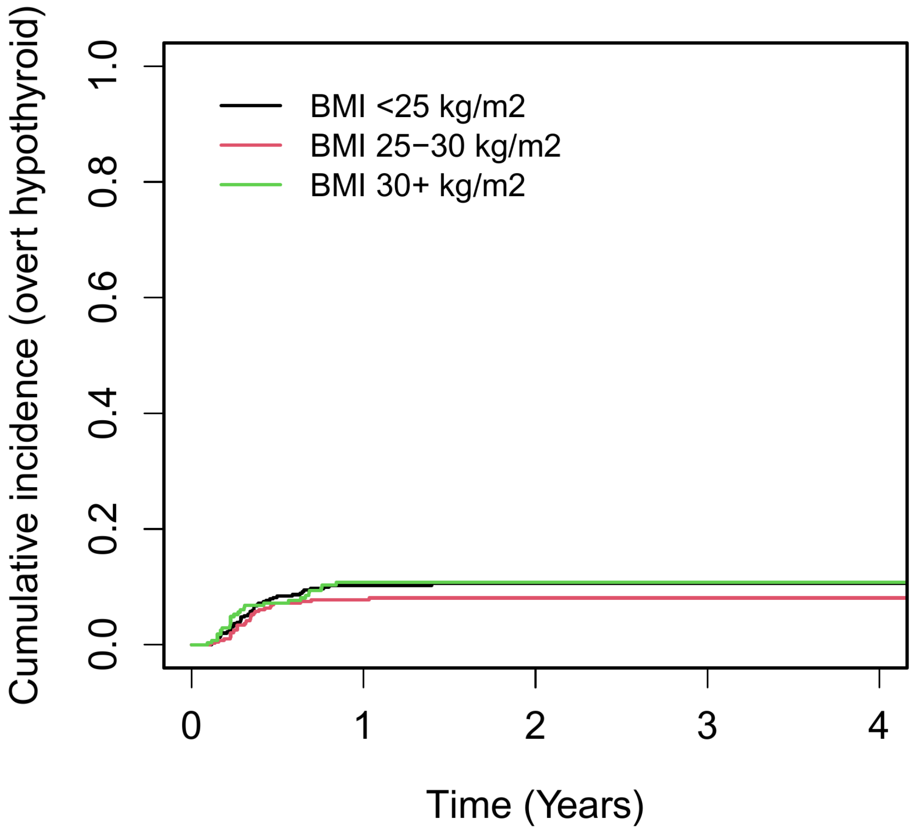

Supplementary figure 15. Cumulative incidence of overt hypothyroidism according to baseline body mass index.

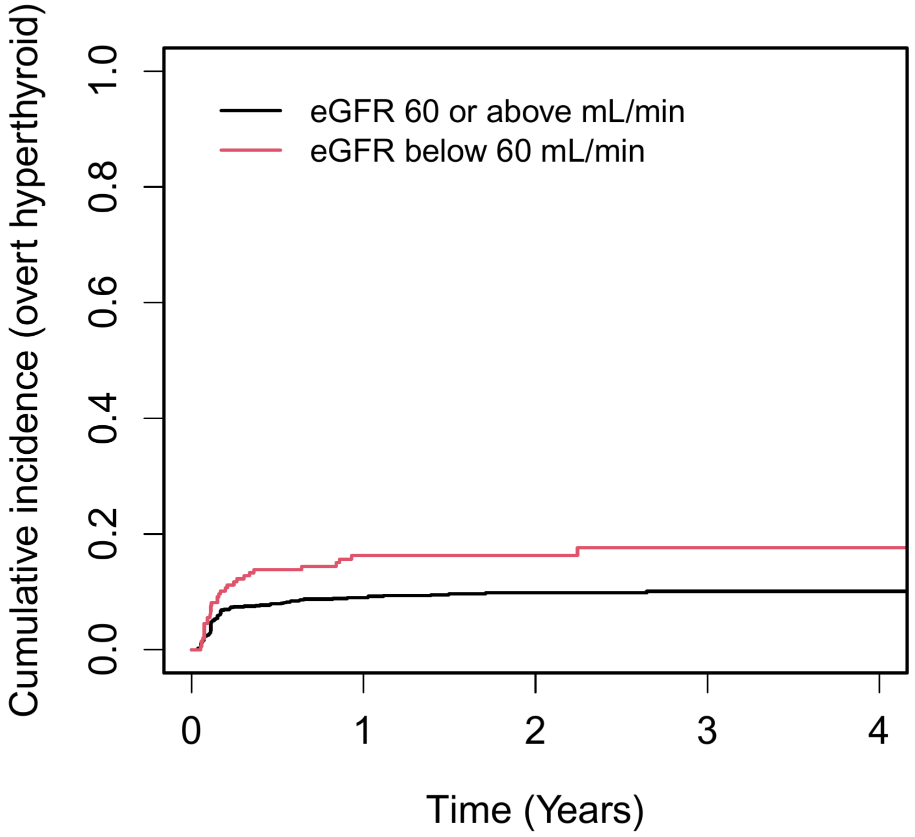

Supplementary figure 16. Cumulative incidence of overt hyperthyroidism according to baseline estimated glomerular filtration rate of 60 or above vs <60 ml/min.

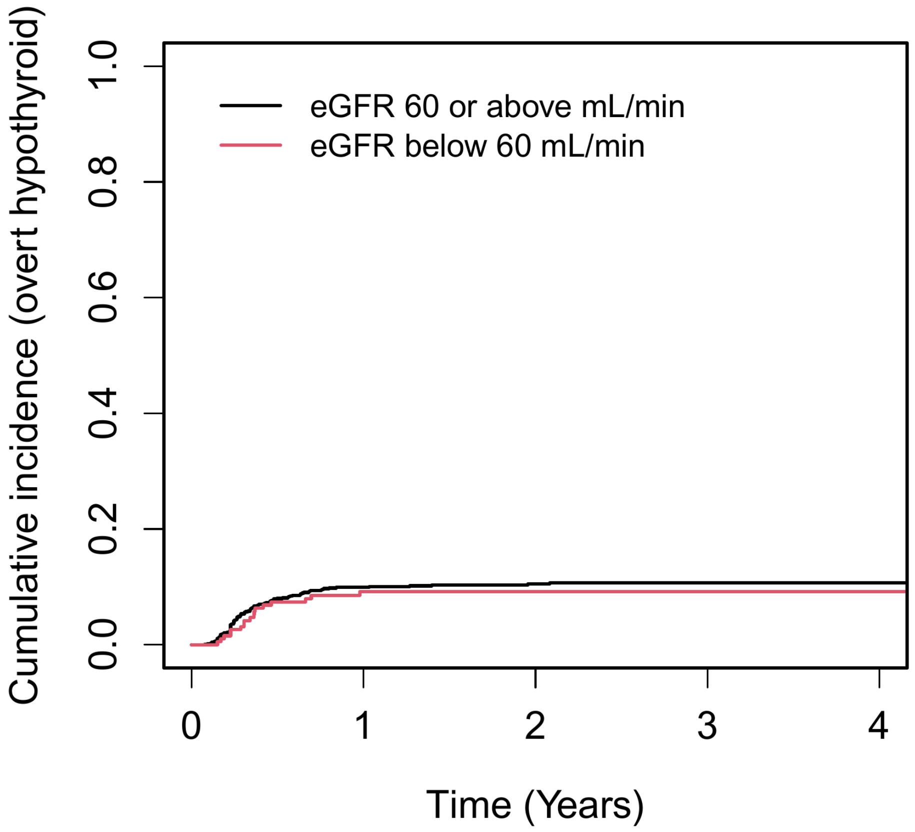

Supplementary figure 17. Cumulative incidence of overt hypothyroidism according to baseline estimated glomerular filtration rate of 60 ml/min or above vs <60 ml/min.

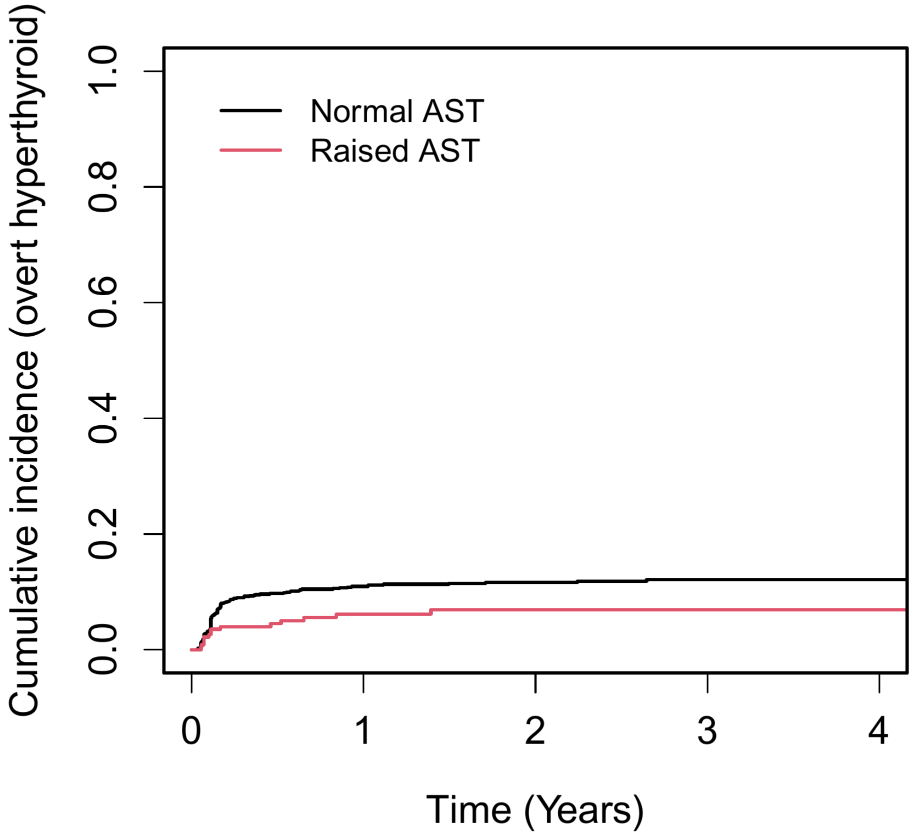

Supplementary figure 18. Cumulative incidence of overt hyperthyroidism according to baseline aspartate aminotransferase concentration.

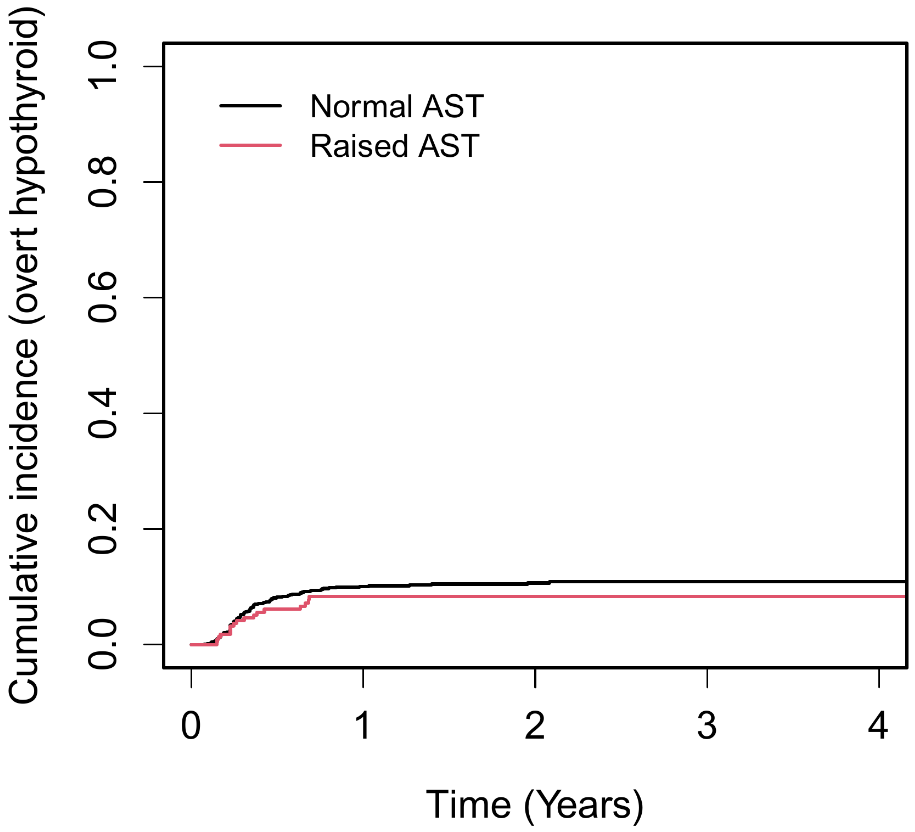

Supplementary figure 19. Cumulative incidence of overt hypothyroidism according to baseline aspartate aminotransferase concentration.

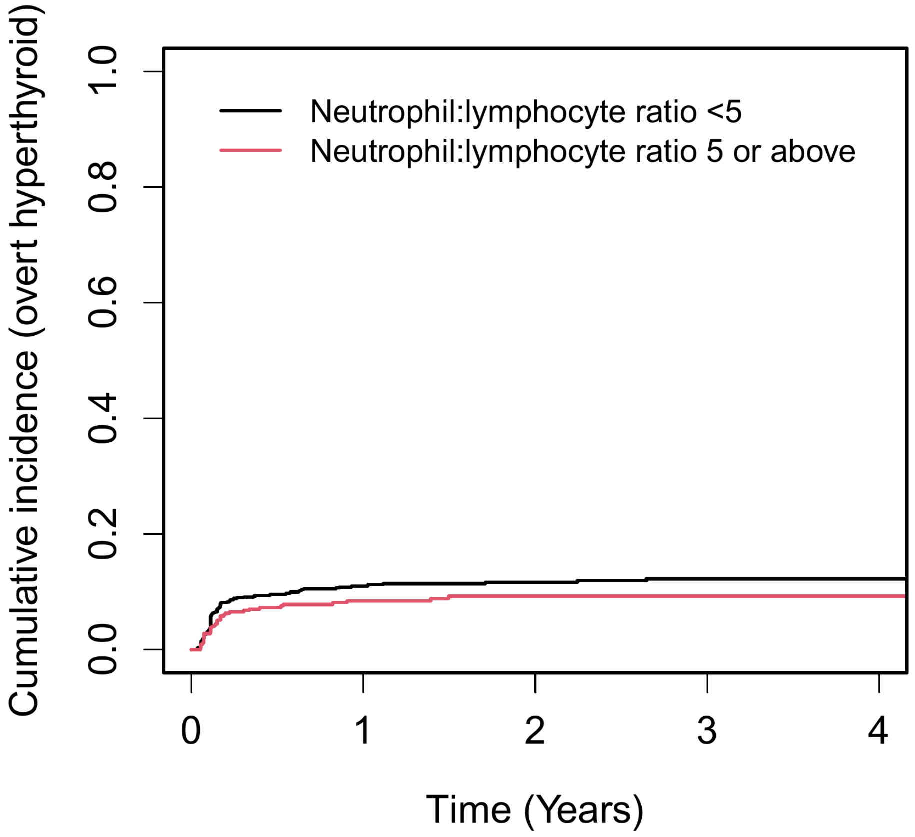

Supplementary figure 20. Cumulative incidence of overt hyperthyroidism according to baseline neutrophil to lymphocyte ratio.

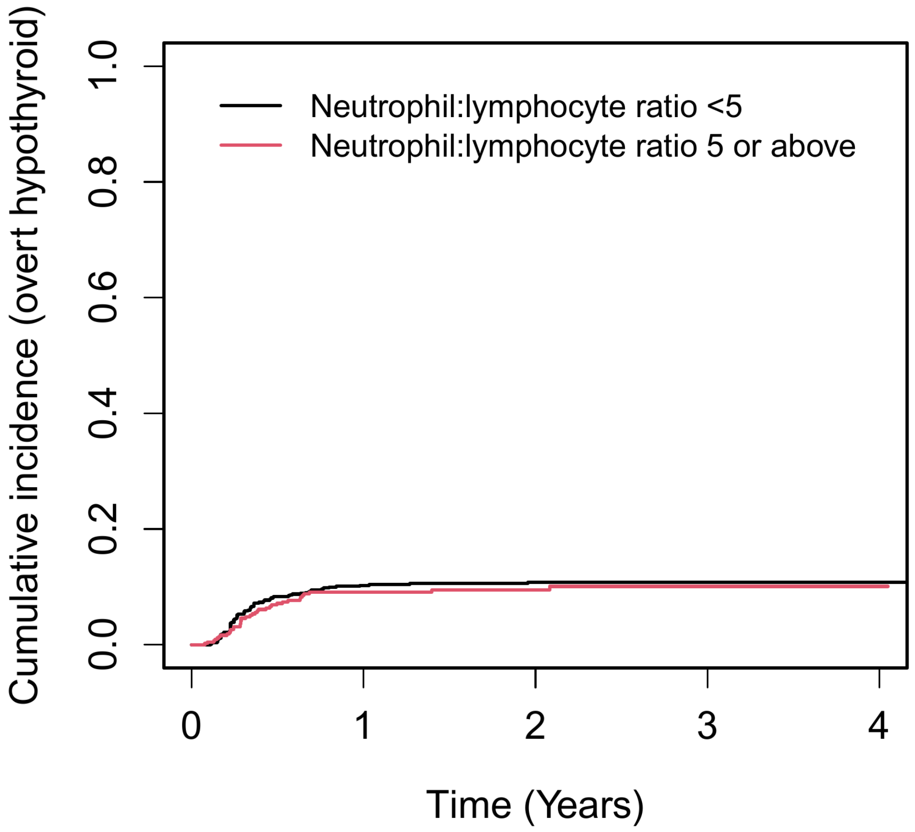

Supplementary figure 21. Cumulative incidence of overt hypothyroidism according to baseline neutrophil to lymphocyte ratio.

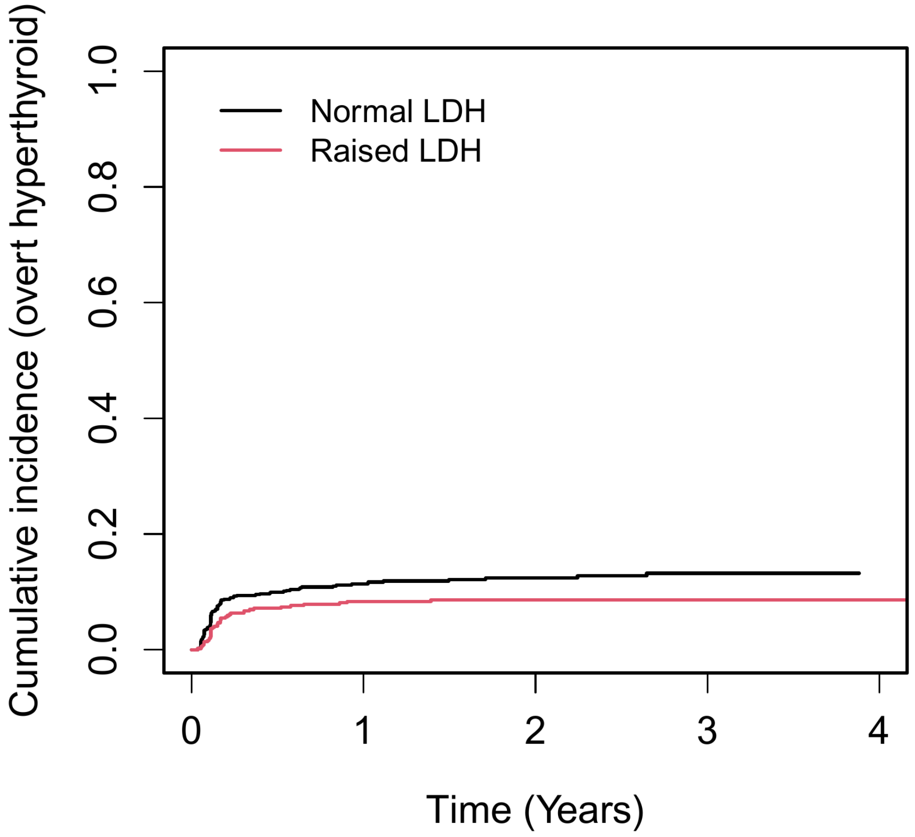

Supplementary figure 22. Cumulative incidence of overt hyperthyroidism according to baseline lactate dehydrogenase concentration.

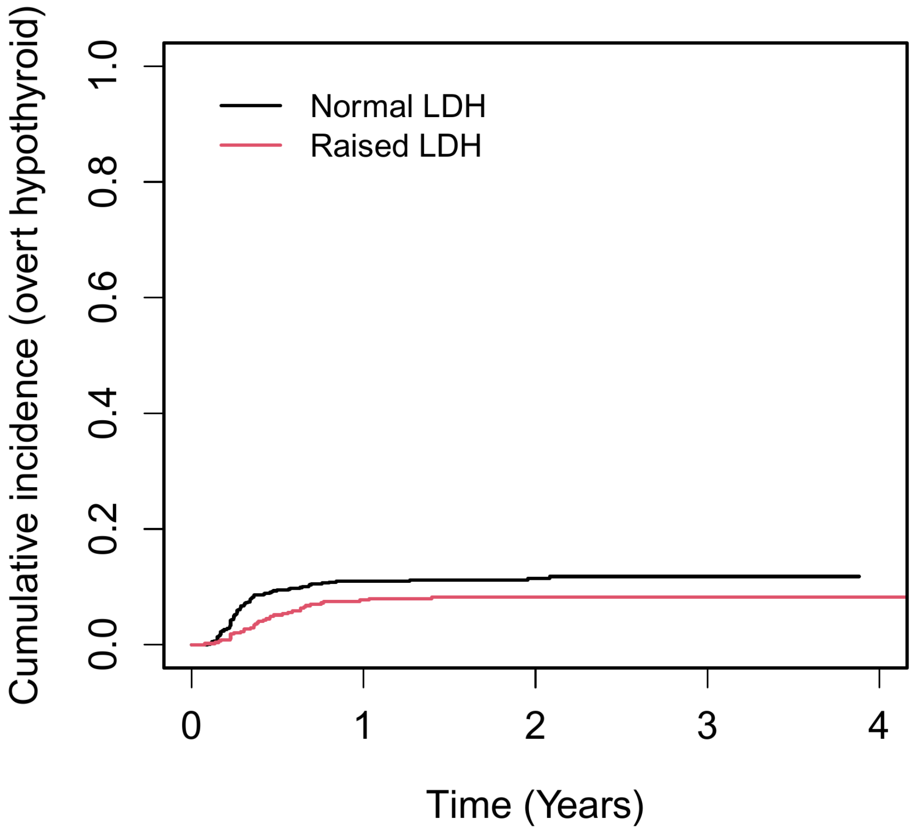

Supplementary figure 23. Cumulative incidence of overt hypothyroidism according to baseline lactate dehydrogenase concentration.

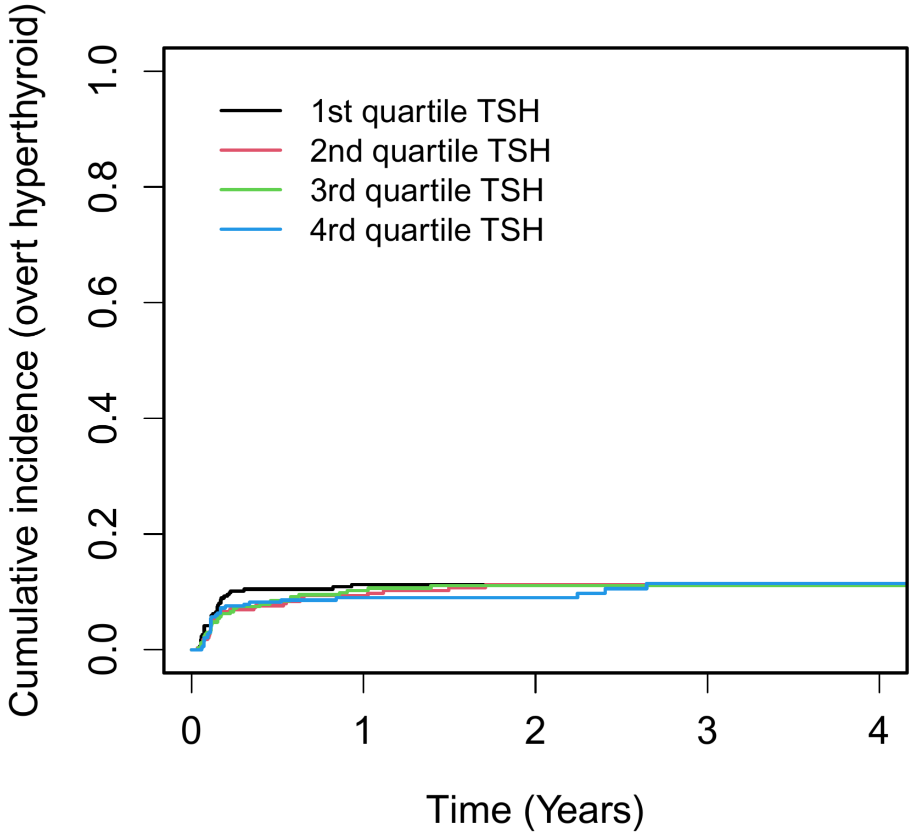

Supplementary figure 24. Cumulative incidence of overt hyperthyroidism according to TSH quartiles.

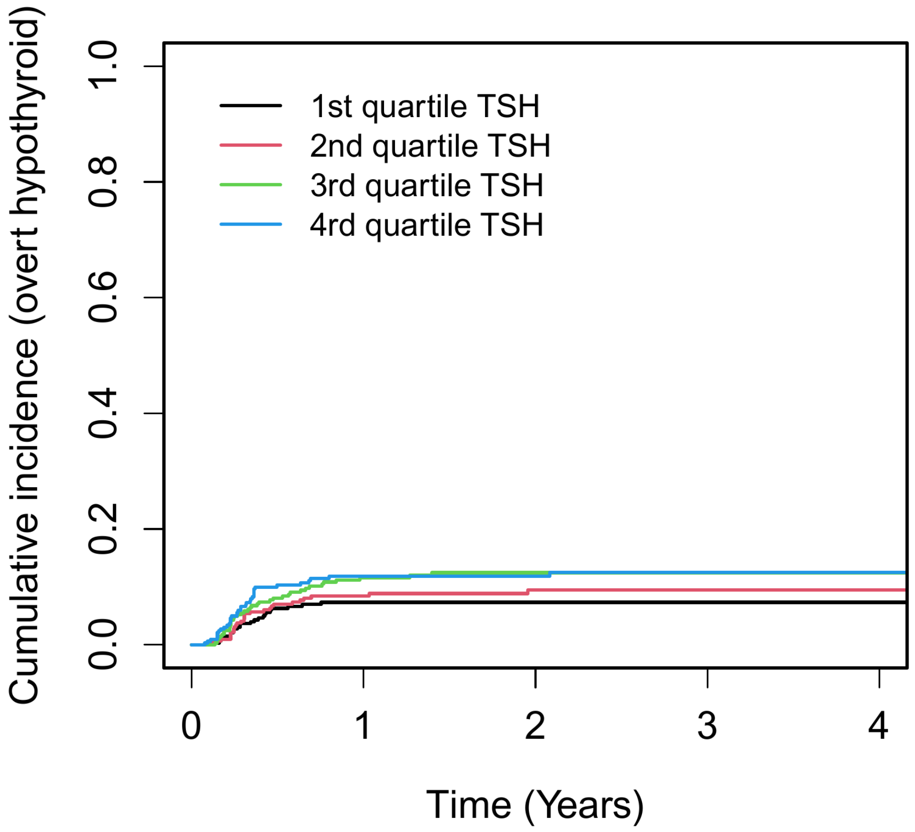

Supplementary figure 25. Cumulative incidence of overt hypothyroidism according to TSH quartiles.

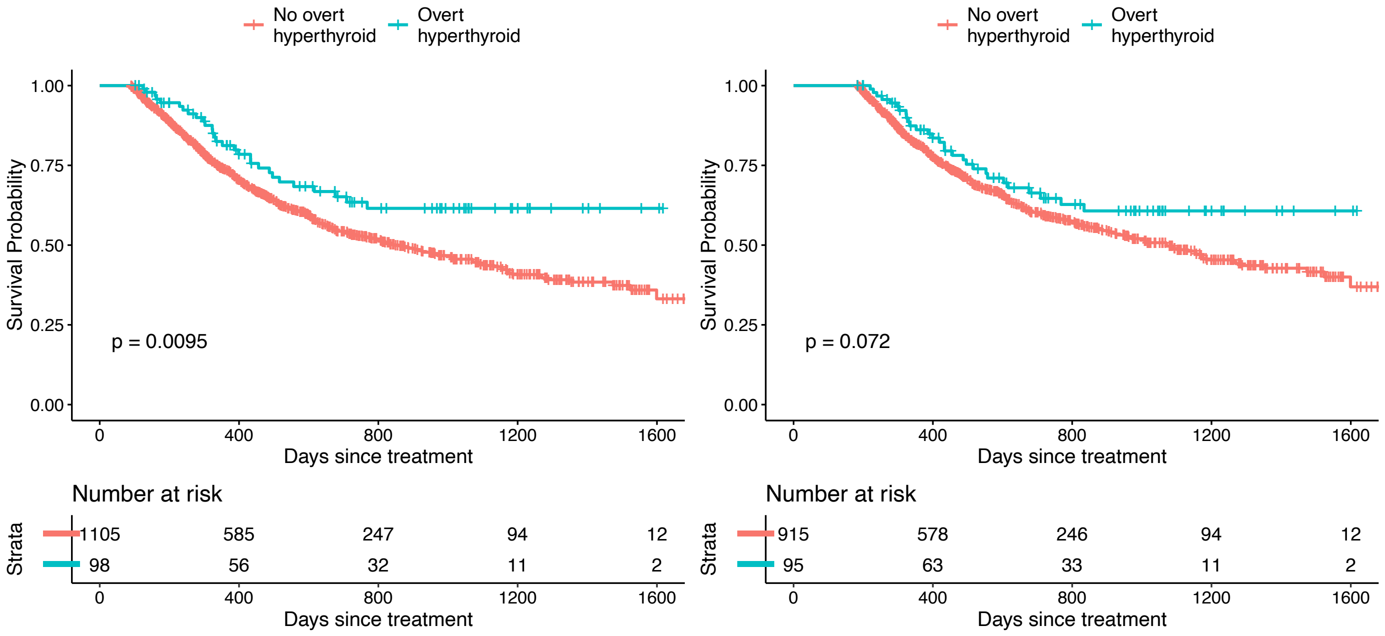

Supplementary figure 26. Kaplan-Meier plots of overall survival according to the presence and absence of overt hyperthyroidism within 3 months (left) and 6 months (right) from treatment initiation.

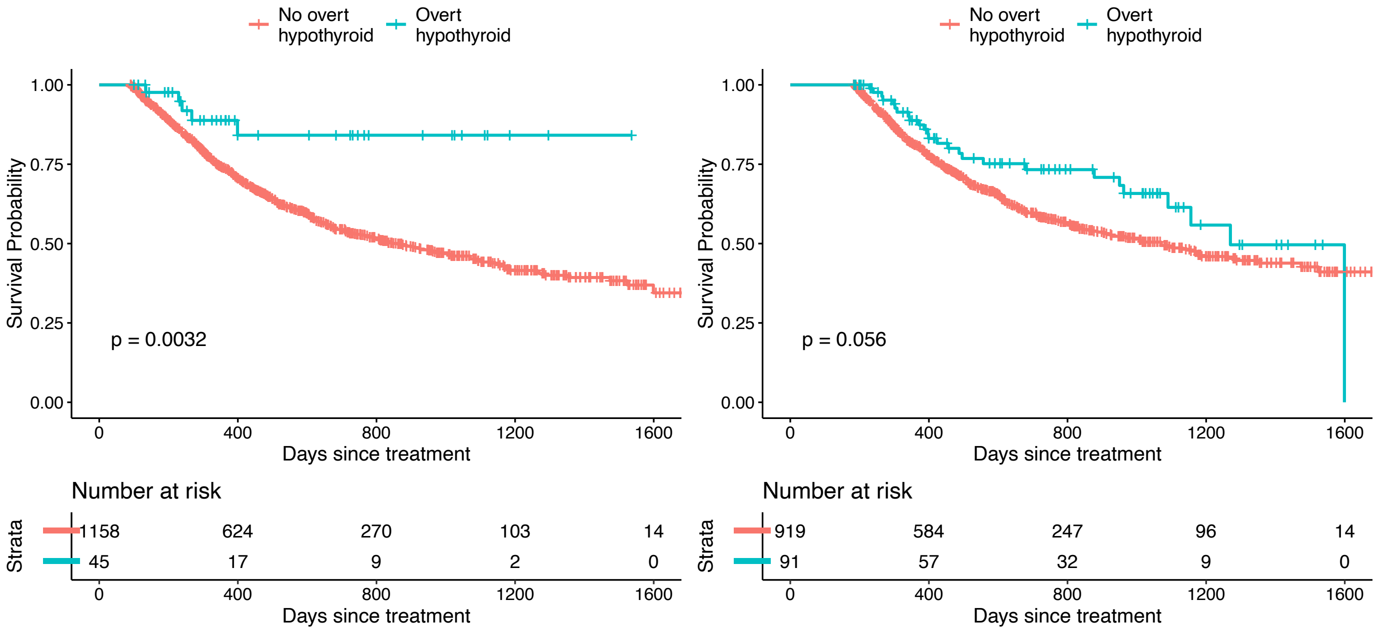

Supplementary figure 27. Kaplan-Meier plots of overall survival according to the presence and absence of overt hypothyroidism within 3 months (left) and 6 months (right) from treatment initiation.

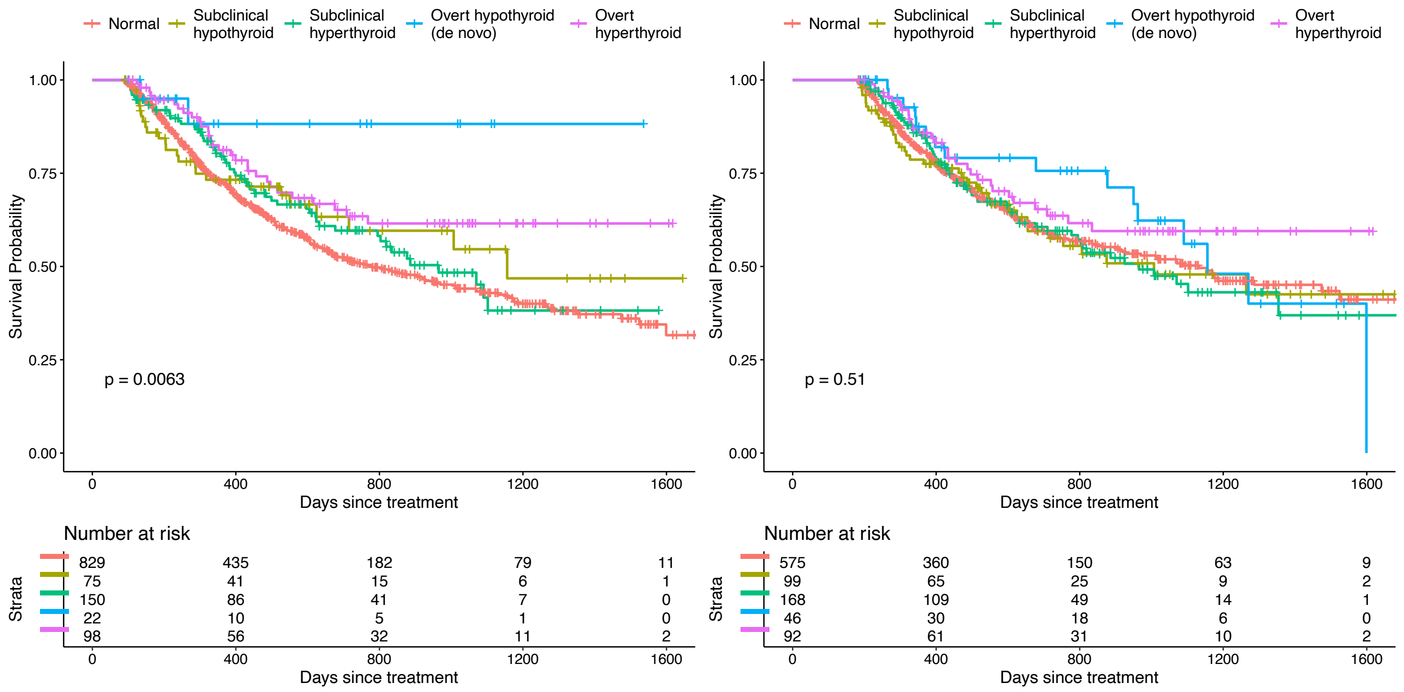

Supplementary figure 28. Kaplan-Meier plots of overall survival by different types of thyroid dysfunction within 3 months (left) and 6 months (right) of treatment initiation.

Supplementary table 1. A table showing the numbers of patients by diagnosis and treatment.

	Lung	Melanoma	Renal	Head, neck and face	Lower gastrointestinal tract	Other
**Pembrolizumab**	125	169	11	36	15	54
**Atezolizumab**	74	≤5	≤5	≤5	≤5	57
**Avelumab**	≤5	≤5	48	≤5	≤5	11
**Chemoimmunotherapy**	131	≤5	≤5	≤5	≤5	62
**Durvalumab**	39	≤5	11	≤5	≤5	≤5
**Ipilimumab + Nivolumab**	≤5	103	101	≤5	≤5	9
**Nivolumab**	≤5	61	16	61	56	32
**Other**	≤5	≤5	≤5	≤5	≤5	42

Supplementary table 2. A table showing proportions of patients with different types of thyroid dysfunction at onset who returned directly to normal thyroid function vs. progressed to other states of dysfunction.

Thyroid dysfunction at onset	Subsequent assessment (median time between first and last TFT)	N (%)
**Subclinical hyperthyroidism (N = 297)**	Normal (6.7 months, IQR: 2.8-15.1 months)	152 (51.2)
	Overt hypothyroidism (12.9 months, IQR: 8.3-23.0)	46 (15.5)
	Overt hyperthyroidism (5.0 months, IQR 2.4-16.6 months)	16 (5.4)
	No further measurements	31 (10.4)
	Subclinical hyperthyroidism (4.2 months, IQR 2.3-11.7 months)	30 (10.1)
	Subclinical hypothyroidism (8.5 months, IQR 1.5-9.8 months)	22 (7.4)
**Subclinical hypothyroidism (N = 210)**	Normal (6.7 months, IQR: 2.8-15.1 months)	76 (36.2)
	Overt hypothyroidism (12.9 months, IQR: 8.3-23.0)	51 (24.3)
	Overt hyperthyroidism (5.0 months, IQR 2.4-16.6 months)	2 (1.0)
	No further measurements	25 (11.9)
	Subclinical hyperthyroidism (4.2 months, IQR 2.3-11.7 months)	52 (24.8)
	Subclinical hypothyroidism (8.5 months, IQR 1.5-9.8 months)	4 (1.9)
**Overt hyperthyroidism (N = 83)**	Normal (6.7 months, IQR: 2.8-15.1 months)	6 (7.2)
	Overt hypothyroidism (12.9 months, IQR: 8.3-23.0)	46 (55.4)
	Overt hyperthyroidism (5.0 months, IQR 2.4-16.6 months)	3 (3.6)
	No further measurements	2 (2.4)
	Subclinical hyperthyroidism (4.2 months, IQR 2.3-11.7 months)	17 (20.5)
	Subclinical hypothyroidism (8.5 months, IQR 1.5-9.8 months)	9 (10.8)

Abbreviations: N (number), TFT (thyroid function test), IQR (interquartile range).

Supplementary table 3. A table showing cumulative incidences of overt hypothyroidism and overt hyperthyroidism by treatment and diagnosis at specific time points from treatment initiation.

	**Overt hyperthyroidism**			**Overt hypothyroidism**		
	**3 months**	**6 months**	**12 months**	**3 months**	**6 months**	**12 months**
All	7.9	8.8	10.0	3.9	7.9	9.7
**Treatment**						
Pembrolizumab	6.2	6.7	7.9	5.3	9.7	12.4
Nivolumab	5.7	6.2	7.6	2.6	6.4	7.8
Ipilimumab + Nivolumab	17.8	18.8	21.0	6.7	12.2	13.8
Chemoimmunotherapy	5.3	7.0	7.7	1.1	3.4	4.8
Atezolizumab	0.8	1.6	3.6	0.7	2.4	4.4
Other	11.6	12.2	12.2	4.4	9.0	10.5
**Diagnosis**						
Lung	5.2	6.0	6.3	2.8	6.3	8.2
Melanoma	10.0	11.0	12.5	5.8	11.0	13.2
Renal	17.9	19.0	20.8	5.9	11.4	13.2
Head, neck and face	5.8	5.8	7.1	4.9	9.4	13.5
Lower gastrointestinal tract	5.2	5.2	6.9	5.3	8.1	9.6
Other	3.8	4.6	6.4	0.8	2.8	3.8

Supplementary table 4. Associations between baseline factors and overt thyroid dysfunction in the first 6 months following immune checkpoint inhibitor initiation among patients who remained on treatment during that time.

	Overt hyperthyroidism				Overt hypothyroidism			
	**No (%)**	**Yes (%)**	**HR (95% CI)***	**p**	**No (%)**	**Yes (%)**	**HR (95% CI)***	**p**
**Sex**								
Female	269 (91.2)	26 (8.8)	1		262 (88.8)	33 (11.2)	1	
Male	463 (89.6)	54 (10.4)	1.08 (0.67 - 1.75)	0.748	474 (91.7)	43 (8.3)	0.64 (0.40 - 1.04)	0.074
**Age (years)**								
<50	89 (89)	11 (11)	1		86 (86)	14 (14)	1	
50-64	262 (86.5)	41 (13.5)	1.10 (0.54 - 2.24)		271 (89.4)	32 (10.6)	0.74 (0.38 - 1.44)	
>65	381 (93.2)	28 (6.8)	0.64 (0.30 - 1.36)	0.493	379 (92.7)	30 (7.3)	0.53 (0.27 - 1.01)	0.105
**BMI kg/m2**								
<25	246 (93.2)	18 (6.8)	1		237 (89.8)	27 (10.2)	1	
25-30	217 (90.8)	22 (9.2)	1.30 (0.67 - 2.49)		220 (92.1)	19 (7.9)	0.88 (0.46 - 1.70)	
30+	148 (88.6)	19 (11.4)	1.43 (0.69 - 2.95)	0.461	153 (91.6)	14 (8.4)	0.82 (0.41 - 1.63)	0.798
**eGFR (mL/min)**								
≥ 60	610 (91.3)	58 (8.7)	1		605 (90.6)	63 (9.4)	1	
< 60	111 (83.5)	22 (16.5)	1.84 (1.00 - 3.39)	0.052	121 (91)	12 (9)	0.96 (0.48 - 1.92)	0.898
**AST**								
Normal AST	604 (89.1)	74 (10.9)	1		615 (90.7)	63 (9.3)	1	
Raised AST	115 (95)	6 (5)	0.46 (0.20 - 1.06)	0.069	110 (90.9)	11 (9.1)	1.20 (0.61 - 2.36)	0.605
**Neutrophil to lymphocyte ratio**								
< 5	528 (89.5)	62 (10.5)	1		533 (90.3)	57 (9.7)	1	
≥ 5	192 (91.4)	18 (8.6)	1.17 (0.65 - 2.12)	0.595	192 (91.4)	18 (8.6)	1.00 (0.54 - 1.85)	0.999
**LDH**								
Normal LDH	454 (88.7)	58 (11.3)	1		459 (89.6)	53 (10.4)	1	
Raised LDH	256 (92.4)	21 (7.6)	0.66 (0.39 - 1.12)	0.124	257 (92.8)	20 (7.2)	0.75 (0.43 - 1.29)	0.299
**TSH quartiles**								
1st	182 (87.9)	25 (12.1)	1		194 (93.7)	13 (6.3)	1	
2nd	189 (92.2)	16 (7.8)	0.55 (0.30 - 1.04)		186 (90.7)	19 (9.3)	1.45 (0.71 - 2.99)	
3rd	188 (90.4)	20 (9.6)	0.70 (0.38 - 1.29)		188 (90.4)	20 (9.6)	1.70 (0.83 - 3.49)	
4th	173 (90.1)	19 (9.9)	0.75 (0.39 - 1.45)	0.142	168 (87.5)	24 (12.5)	2.01 (0.99 - 4.05)	0.075

Abbreviations: HR (hazard ratio), CI (confidence interval), BMI (body mass index), eGFR (estimated glomerular filtration rate), LDH (lactate dehydrogenase), TSH (thyroid stimulating hormone). *adjusted for age, sex, performance status, setting and immune checkpoint inhibitor.

Supplementary table 5. HRs for the associations of treatment indication with overt hypothyroidism and overt hyperthyroidism.

**Indication**	**Total**	**Overt hypothyroid (N)**	**HR 95% CI hyperthyroid**	**Overt hyperthyroidism (N)**	**HR 95% CI hypothyroidism**
Pembrolizumab or atezolizumab monotherapy for lung cancer (advanced)	199	20	1	10	1
Chemoimmunotherapy for lung cancer (advanced)	130	6	1.18 (0.46-3.01)	8	0.47 (0.19-1.16)
Pembrolizumab or nivolumab for melanoma (adjuvant)	124	18	2.16 (0.96-4.89)	15	1.37 (0.72-2.63)
Pembrolizumab or nivolumab for melanoma (advanced)	105	9	1.75 (0.69-4.43)	8	0.97 (0.44-2.12)
Ipilimumab + nivolumab for melanoma (advanced)	103	15	4.11 (1.87-9.03)	21	1.47 (0.73-2.96)
Ipilimumab + nivolumab for renal cancer (advanced)	101	14	5.04 (2.40-10.60)	25	1.43 (0.71-2.87)
Pembrolizumab or nivolumab for head and neck cancer (advanced)	97	10	1.19 (0.43-3.31)	6	1.24 (0.56-2.75)
Other adjuvant treatment	74	5	1.97 (0.74-5.29)	7	0.78 (0.29-2.10)
Pembrolizumab or nivolumab for LGI cancer (advanced)	71	7	1.53 (0.55-4.27)	6	0.93 (0.39-2.24)
Other Chemoimmunotherapy (advanced cancer)	57	2	1.88 (0.68-5.21)	6	0.31 (0.07-1.33)
Other immunotherapy (advanced cancer)	288	20	2.04 (0.98-4.24)	27	0.78 (0.42-1.43)

Abbreviations: HR (hazard ratio), CI (confidence interval), LGI (lower gastrointestinal). *adjusted for age, sex and performance status.

Supplementary table 6. A table showing the characteristics of patients included and excluded from the 3-month and 6-month landmark analyses of the associations of overt thyroid dysfunction (vs. no overt thyroid dysfunction) with overall survival.

	**3-month landmark**		**6-month landmark**	
	**Excluded (%)**	**Included (%)**	**Excluded (%)**	**Included (%)**
**N**	146	1203	339	1010
**Sex**				
Female	53 (36)	440 (37)	121 (36)	372 (37)
Male	93 (64)	763 (63)	218 (64)	638 (63)
**Age**				
Median (IQR)	64.0 (18.9)	65.4 (16.8)	65.9 (18.2)	65.1 (16.7)
**ECOG performance status**				
0	63 (43)	710 (59)	158 (47)	615 (61)
1	54 (37)	306 (25)	120 (35)	240 (24)
2+	12 (8)	31 (3)	17 (5)	26 (3)
Unknown	17 (12)	156 (13)	44 (13)	129 (13)
**Overt thyroid dysfunction before the landmark**				
No	138 (95)	1083 (90)	311 (92)	872 (86)
Yes	8 (5)	120 (10)	28 (8)	138 (14)
**Reasons for exclusion**				
Censored before landmark	72 (49)	0 (0)	159 (47)	0 (0)
Died before landmark	74 (51)	0 (0)	180 (53)	0 (0)
**Setting**				
Adjuvant	12 (8)	186 (15)	27 (8)	171 (17)
Advanced	134 (92)	1017 (85)	312 (92)	839 (83)
**ICI**				
Pembrolizumab	44 (30)	365 (30)	98 (29)	311 (31)
Nivolumab	29 (20)	201 (17)	68 (20)	162 (16)
Ipilimumab + Nivolumab	17 (12)	198 (16)	43 (13)	172 (17)
Chemoimmunotherapy	21 (14)	173 (14)	56 (17)	138 (14)
Atezolizumab	22 (15)	113 (9)	46 (14)	89 (9)
Other	13 (9)	153 (13)	28 (8)	138 (14)
**Diagnosis**				
Lung	38 (26)	335 (28)	91 (27)	282 (28)
Melanoma	25 (17)	307 (26)	60 (18)	272 (27)
Renal	12 (8)	179 (15)	25 (7)	166 (16)
Head, neck and face	26 (18)	79 (7)	51 (15)	54 (5)
Lower gastrointestinal tract	11 (8)	67 (6)	29 (9)	49 (5)
Other	34 (23)	236 (20)	83 (24)	187 (19)

Abbreviations: N (number), IQR (interquartile range), ECOG (Eastern Cooperative Oncology Group), ICI (immune checkpoint inhibitor)

Supplementary table 7. Associations between overt thyroid dysfunction and overall survival estimated using standard Cox regression, Cox regression with a time dependent variable for thyroid status and landmark analyses.

**Presence / absence**	**HR* (95% CI) standard Cox model**	**HR* (95% CI) time dependent Cox model**	**HR* (95% CI) 3-m landmark**	**HR* (95% CI) 6-m landmark**
Overt thyroid dysfunction	0.65 (0.50-0.85, p=0.001)	0.79 (0.60-1.03, p=0.084)	0.74 (0.51-1.07 p=0.108)	0.91 (0.66-1.24 p=0.540)
Overt hyperthyroidism	0.72 (0.52-0.99, p=0.043)	0.88 (0.64-1.21, p=0.435)	0.86 (0.58-1.26 p=0.438)	0.95 (0.65-1.41 p=0.809)
Overt hypothyroidism	0.57 (0.40-0.80, p=0.001)	0.73 (0.51-1.03, p=0.069)	0.40 (0.17-0.98 p=0.045)	0.85 (0.57-1.27 p=0.428)

Abbreviations: HR (hazard ratio), CI (confidence interval), m (months). *adjustments were made for age, sex, performance status, setting, immune checkpoint inhibitor and cancer type.

Supplementary table 8. Associations between different types of thyroid dysfunction and overall survival estimated using Cox regression and landmark analyses.

**Thyroid status**	**HR* (95% CI) standard Cox model**	**HR* (95% CI) 3-m landmark**	**HR* (95% CI) 6-m landmark**
Normal thyroid function	1. (Ref.)	1. (Ref.)	1. (Ref.)
Subclinical hypothyroidism	0.73 (0.56-0.94 p=0.017)	0.84 (0.55-1.29 p=0.425)	1.15 (0.81-1.64 p=0.438)
Subclinical hyperthyroidism	0.81 (0.64-1.01 p=0.064)	0.91 (0.68-1.20 p=0.500)	1.10 (0.83-1.46 p=0.508)
Overt hypothyroidism (de novo)	0.53 (0.34-0.82 p=0.004)	0.24 (0.06-0.99 p=0.048)	0.83 (0.49-1.40 p=0.487)
Overt hyperthyroidism	0.62 (0.44-0.87 p=0.005)	0.81 (0.55-1.20 p=0.302)	1.02 (0.69-1.53 p=0.909)

Abbreviations: HR (hazard ratio), CI (confidence interval), m (months). *adjustments were made for age, sex, performance status, setting, immune checkpoint inhibitor and cancer type.

Supplementary table 9. HRs for the associations between treatment indication and OS.

Indication	Total	Deaths	HR (95% CI) univariable	HR (95% CI) multivariable*
Pembrolizumab or atezolizumab monotherapy for lung cancer (advanced)	199	109	1	1
Chemoimmunotherapy for lung cancer (advanced)	130	72	1.60 (1.18-2.16)	1.62 (1.20-2.19)
Pembrolizumab or nivolumab for melanoma (adjuvant)	124	11	0.13 (0.07-0.24)	0.14 (0.08-0.26)
Pembrolizumab or nivolumab for melanoma (advanced)	105	41	0.69 (0.48-0.99)	0.65 (0.45-0.93)
Ipilimumab + nivolumab for melanoma (advanced)	103	29	0.49 (0.33-0.74)	0.52 (0.34-0.79)
Ipilimumab + nivolumab for renal cancer (advanced)	101	33	0.50 (0.34-0.74)	0.52 (0.35-0.77)
Pembrolizumab or nivolumab for head and neck cancer (advanced)	97	49	1.81 (1.29-2.54)	1.90 (1.34-2.70)
Other adjuvant treatment	74	17	0.46 (0.27-0.76)	0.46 (0.27-0.78)
Pembrolizumab or nivolumab for LGI cancer (advanced)	71	46	1.76 (1.25-2.49)	1.94 (1.36-2.77)
Other Chemoimmunotherapy (advanced cancer)	57	25	0.92 (0.59-1.41)	0.98 (0.62-1.52)
Other immunotherapy (advanced cancer)	288	130	0.91 (0.71-1.17)	0.86 (0.66-1.11)

Abbreviations: HR (hazard ratio), CI (confidence interval), LGI (lower gastrointestinal). *adjusted for age, sex and performance status.

Supplementary information relating to measurement of free T4 (fT4) and thyroid stimulating hormone (TSH). The local laboratory reference range for fT4 was 10 - 22 pmol/L and for TSH was 0.55 - 4.78 mU/L. TFT assays were performed on the Siemens Centaur XP analyser (Siemens, Camberley, UK). TSH and free T4 assays were all enrolled on the RIQAS international external quality assessment scheme (Randox Laboratories International, Crumlin, UK) for the duration of this study to ensure robust quality management of the laboratory test results.
